# The Use of Task Shifting to Improve Treatment Engagement in an Internet-Based Mindfulness Intervention Among Chinese University Students: Randomized Controlled Trial

**DOI:** 10.2196/25772

**Published:** 2021-10-13

**Authors:** Marcus Rodriguez, Tory A Eisenlohr-Moul, Jared Weisman, M Zachary Rosenthal

**Affiliations:** 1 Department of Psychology Pitzer College Claremont, CA United States; 2 Boston Child Study Center Los Angeles, CA United States; 3 Neuropsychiatric Institute Department of Psychiatry University of Illinois at Chicago College of Medicine Chicago, IL United States; 4 Pitzer College Claremont, CA United States; 5 MCR Labs, LLC Framingham, MA United States; 6 Department of Psychology & Neuroscience Duke University Durham, NC United States

**Keywords:** mindfulness, mental health, social support, internet-based intervention, treatment outcome, university students, smartphone, mobile phone

## Abstract

**Background:**

Traditional in-person psychotherapies are incapable of addressing global mental health needs. Use of computer-based interventions is one promising solution for closing the gap between the amount of global mental health treatment needed and received.

**Objective:**

Although many meta-analyses have provided evidence supporting the efficacy of self-guided, computer-based interventions, most report low rates of treatment engagement (eg, high attrition and low adherence). The aim of this study is to investigate the efficacy of an adjunctive treatment component that uses task shifting, wherein mental health care is provided by nonspecialist peer counselors to enhance engagement in an internet-based, self-directed, evidence-based mindfulness intervention among Chinese university students.

**Methods:**

From 3 universities across China, 54 students who reported at least mild stress, anxiety, or depression were randomly assigned to a 4-week internet-based mindfulness intervention (MIND) or to the intervention plus peer counselor support (MIND+), respectively. *Be Mindful* delivers all the elements of mindfulness-based cognitive therapy in an internet-based, 4-week course. Participants completed daily monitoring of mindfulness practice and mood, as well as baseline and posttreatment self-reported levels of depression, anxiety, stress, and trait mindfulness. We screened 56 volunteer peer counselor candidates who had no former training in the delivery of mental health services. Of these, 10 were invited to participate in a day-long training, and 4 were selected. Peer counselors were instructed to provide 6 brief (15-20 minute) *sessions* each week, to help encouraging participants to complete the internet-based intervention. Peer counselors received weekly web-based group supervision.

**Results:**

For both conditions, participation in the internet-based intervention was associated with significant improvements in mindfulness and mental health outcomes. The pre-post effect sizes (Cohen *d*) for mindfulness, depression, anxiety, and stress were 0.55, 0.95, 0.89, and 1.13, respectively. Participants assigned to the MIND+ (vs MIND) condition demonstrated significantly less attrition and more adherence, as indicated by a greater likelihood of completing posttreatment assessments (16/27, 59% vs 7/27, 26%; χ^2^_1_=6.1; *P*=.01) and a higher percentage of course completion (72.6/100, 72.6% vs 50.7/100, 50.7%; *t*_52_=2.10; *P*=.04), respectively. No significant between-group differences in daily frequency and duration of mindfulness practice were observed. Multilevel logistic growth models showed that MIND+ participants reported significantly greater pre-post improvements in daily stress ratings (interaction estimate 0.39, SE 0.18; *t*_317_=2.29; *P*=.02) and depression (interaction estimate 0.38, SE 0.16; *t*_330_=2.37; *P*=.02) than those in the MIND condition.

**Conclusions:**

This study provides new insights into effective ways of leveraging technology and task shifting to implement large-scale mental health initiatives that are financially feasible, easily transportable, and quickly scalable in low-resource settings. The findings suggest that volunteer peer counselors receiving low-cost, low-intensity training and supervision may significantly improve participants’ indices of treatment engagement and mental health outcomes in an internet-based mindfulness intervention among Chinese university students.

## Introduction

### Background

Approximately 1 in 5 adults in the United States experiences mental illness annually [1], with young adults (aged 18-25 years) reporting the highest prevalence. In economic terms, these problems cost the United States more than US $193.2 billion each year, both in direct (eg, treatment) and indirect (ie, productivity loss at workplace, school, and home) expenses [2]. Globally, 3 out of 4 people report that they prefer therapy to psychopharmacology; however, despite the existence of empirically supported behavioral interventions targeting serious mental illness conditions, nearly two-third of US adults with these conditions do not receive services [3,4]. In short, there is a large gap between treatment needed and treatment received for mental disorders, and the mental health treatment gap is larger in low- and middle-income countries (LMICs) than in higher-income countries [5]. In China, more than 1 in 4 university students reported being depressed; moreover, although 4 out of 10 people worldwide suffer from a psychiatric illness at some point in their lives, nearly half of the world’s population lives in a country with less than 1 psychiatrist per 100,000 people [6,7]. The reasons for these treatment gaps are multifaceted and include factors such as stigma, perceived helpfulness of treatments, and convenience. However, insufficient human resources and mental health infrastructure are arguably the greatest contributors.

Traditional psychotherapy training models (ie, 2-7 years of graduate school) and psychotherapies (one-on-one and in-person sessions) are incapable of singularly closing the global mental health gap in LMICs. Fundamentally, new approaches are needed to increase access to effective mental health care in an economic, feasible, and scalable manner to address global needs [8], and computer- and web-based interventions are promising solutions to closing the large gap between mental health treatment required and treatment received. In addition to addressing concerns of physical accessibility to treatment providers, web-based interventions accessed privately from the household allow individuals to avoid the perceived stigma associated with seeking mental health services [9]. Although not every country prioritizes the enhancement of their mental health infrastructure, most countries prioritize the development of their telecommunications infrastructure.

### Mindfulness- and Acceptance-Based Interventions

Mindfulness- and acceptance-based interventions have been successfully used to address clinical dysfunction across a range of physical and mental health disorders. Specifically, systematic reviews and meta-analyses have demonstrated mindfulness and acceptance to be beneficial in treating physical health conditions, such as chronic pain [10], as well as mental health problems including depression [11,12], substance use disorders [13], eating disorders [14], anxiety [12,15], stress [16], and general psychological health [17]. A recent meta-analysis explored the efficacy of mindfulness-based interventions delivered through the internet [18]. Overall pre-post, between-group effects (Hedge *g*) were reported for stress (*g*=0.51), depression (*g*=0.29), anxiety (*g*=0.22), well-being (*g*=0.23), and mindfulness (*g*=0.32), all with nominal statistical significance thresholds of *P*<.50 [18]. Overall, the results of this meta-analysis provide promising initial evidence for the efficacy of mindfulness-based interventions delivered through the internet.

### Existing Barriers to Treatment

Although technology-based mindfulness interventions can be effective, treatment engagement is a key barrier to the implementation of these approaches. High attrition and low adherence rates are commonly observed in research and practice. Nonadherence can diminish the effectiveness of interventions [19-21]. For example, although *PTSD Coach*, an app developed and used by the Department of Veteran’s Affairs, was downloaded more than 150,000 times, fewer than 15% of the people used it within 1 week [22].

Adherence is especially relevant in mindfulness training because regular practice is thought to be essential for developing mindfulness skills. In their meta-analytic review of web-based mindfulness interventions, Spijkerman et al [18] reported adherence rates between 39.5% and 92%, when adherence was deﬁned as completion of all sessions. However, adherence rates were only reported for 33% (5/15) of the studies that were included in the review.

Although many web-based mindfulness interventions have been shown to be effective, about half of the published studies did not report treatment engagement outcomes, and of those that did, most reported low engagement (high attrition and low adherence). Researchers have repeatedly noted that in spite of the effectiveness of web-based interventions, it is a consistent challenge to reliably measure the amount and quality of mindfulness practice with which people engage [23,24]. Moreover, previous approaches to improve the scale and scope of mental health care systems in LMICs have largely failed because of budgetary constraints, lack of cost estimates, and logistical breakdowns, inhibiting the development of suitable infrastructure [25]. Novel, feasible, and scalable approaches are needed to not only improve treatment implementation but also treatment engagement. As an emerging platform for the provision of mental health interventions, web-based interventions need more quantitative data to evaluate factors associated with treatment efficacy, as well as the feasibility and acceptability of these approaches. Treatment attrition and adherence rates are two important indices of treatment feasibility that can be assessed in mobile interventions and correlated with factors that can be used to promote patient retention. Given the axiom that the development of viable mindfulness skills requires discipline and regular practice, further research is needed to explore the existing barriers to adherence to develop strategies for enhancing treatment engagement.

### Existing Research

Previous research indicates that providing therapist support has a positive inﬂuence on adherence and enhances the effectiveness of web-based psychological interventions [26-28]. Spijkerman et al [18] reported larger effect sizes for mindfulness and stress for web-based mindfulness interventions with therapist guidance (*g*=0.89 and *g*=0.43, respectively), than for those interventions without guidance (*g*=0.19 and *g*=0.22, respectively). Although offering therapist guidance may potentially improve adherence and treatment outcomes, it is costly and may restrict the scalability of the intervention, particularly in LMICs. To overcome these barriers, some web-based interventions provide automated support, which has preliminary evidence supporting its efficacy [29-31]. For example, Oinas-Kukkonen and Harjumaa [32] suggest that system design and automated support may even be as effective as human support.

### Task Sharing

Task sharing or *task shifting*, wherein mental health care is provided by nonspecialist peer counselors (eg, nurses, clergymen, teachers, community leaders), has recently been investigated as a promising strategy to overcome human resource shortages in LMICs. In this model of care, peer counselors receive training, supervision, and oversight from mental health care professionals (eg, psychiatrists, psychologists, and clinical social workers). Task shifting has been shown to be effective in the treatment of mental health problems [33,34], and in enhancing treatment engagement (eg, HIV medication compliance [35], child and maternal health care [36], and noncommunicable disease management [including depression] [37]). Thus, a task-shifting model shows promise as an alternative strategy to therapist guidance or peer support for enhancing treatment engagement and outcomes in self-guided, web-based mindfulness interventions.

### Objectives

The aim of this study is to examine whether an adjunctive, task-shifting component (MIND+) enhances treatment engagement in a mindfulness intervention for stress and depression among Chinese undergraduate and graduate students. Individuals were randomly assigned to a brief (4-week), self-guided, web-based, mindfulness intervention (MIND), or the intervention plus support from nonspecialist peer counselors (MIND+). Peer counselors were instructed to engage in brief (15-20 minutes) weekly meetings with MIND+ participants via text or phone call during the course of treatment, with the intention of supporting and encouraging participants to complete the internet-based intervention. It was hypothesized that at posttreatment, participants randomly assigned to MIND+ (vs those assigned to MIND) would show (1) less attrition (higher completion rates of assessment), (2) greater adherence (higher percentage of course completion), (3) greater reductions in stress and depression levels, and (4) greater increases in mindfulness.

## Methods

### Participant Overview

Participants were 54 currently enrolled university students (undergraduate, master’s, and doctoral programs) from 36 universities across China. Their mean age was 23.5 years (SD 3.17), and 74% (40/54) identified as female. Out of the 54 participants, 29 (54%) were master’s students, 21 (39%) were undergraduate students, and 4 (7%) were doctoral students. All participants reported having passed an English proficiency test: 11% (6/54) reported passing the College English Test (CET; level 4); 72% (39/54), the CET (level 6); and 17% (9/54), the Test of English as a Foreign Language. All participants denied currently receiving formal mental health treatment; 80% (43/54) reported no history of mental health treatment, 11% (6/54) reported formerly receiving therapy, 4% (2/54) reported formerly receiving medication, and 6% (3/54) reported formerly receiving both treatment and medication. Out of the 54 participants, 47 (87%) reported no previous mindfulness training, and 3 (6%) reported practicing mindfulness meditation in the past year.

### Participant Recruitment

Participants were recruited via WeChat blogs, student club listservs, and university websites’ listing of available jobs and research opportunities. Interested individuals completed a web-based screening assessment. Eligible students were contacted by the study coordinator, who conducted phone interviews and orientation to the study procedures. Students who provided proof of student status and emergency contact information received a link to the baseline assessment measures. Students who completed the web-based baseline assessment measures were randomly assigned to a brief, 4-week internet-based mindfulness intervention (MIND), or to the intervention plus peer counselor support (MIND+). The inclusion and exclusion criteria have been provided in Textbox 1.

**Participant inclusion and exclusion criteria.**

**Inclusion criteria**

- Is currently enrolled in a university in China (undergraduate, graduate, or doctoral)
- Has a smartphone and regular access to the internet
- Demonstrates the ability to read and understand Mandarin
- Reports passing at least College English Test (level 4)
- Experiences at least mild depression and anxiety

**Exclusion criteria**

- Is aged <18 years
- Does not provide proof of current student status and emergency contact
- Currently experiences manic or psychotic symptoms
- Expresses suicidal or homicidal ideation during the intake phone interview

### Peer Counselor Overview

#### Overview

Peer counselors included 4 currently enrolled female students at 3 different universities in Beijing. At the time of recruitment, their mean age was 27.5 years (SD 6.8), including 1 undergraduate (psychology), 1 master’s (business), and 2 doctoral (nursing) students. None of the peer counselors reported formal training or experience in mindfulness practice or the provision of mental health services. All participants reported having passed at least the CET (level 6).

#### Peer Counselor Recruitment

Web-based advertisements were posted on university research, student club, and mindfulness listservs. A total of 56 candidates responded to the web-based survey, expressing interest in participating in the study as peer counselors. Those who met the inclusion criteria were contacted via telephone to screen for the exclusion criteria and confirm their understanding of the study and willingness to participate in the in-person training and orientation. Volunteer peer counselor candidates who met all the inclusion criteria were invited to the in-person training and orientation. After this training, participants were contacted via telephone to once again assess their willingness to engage in the study. We selected 4 individuals as peer counselors based on their English proficiency, reported level of enthusiasm for the project, and the researchers’ assessment of their nonspecific factors. Each individual was given access to the internet-based intervention and a 6-week period to complete the course. After completing the course, peer counselors were paired with study participants who were randomized to the MIND+ group. The inclusion and exclusion criteria for peer counselors have been illustrated in Textbox 2.

**Peer counselor inclusion and exclusion criteria.**

**Inclusion criteria**

- Is currently enrolled in a university in Beijing (undergraduate, graduate, or doctoral)
- Has a smartphone and regular access to the internet
- Demonstrates the ability to read and communicate in Mandarin and English
- Is willing to provide brief (15-20 minute) peer-support chats per week per participant
- Is willing to participate in web-based group supervision for 1 hour per week
- Is willing to complete the internet-based mindfulness intervention

**Exclusion criteria**

- Is aged <18 years
- Reports previous or current format training in mindfulness or psychotherapy
- Reports current treatment (psychotherapy or medication) for a mental health problem
- Is unable to attend the day-long, in-person training in Beijing

#### Peer Counselor Training

The in-person training took place for 8 hours in Beijing. All lectures and discussions were conducted in Mandarin. Peer counselor candidates listened to lectures on topics related to peer counseling and the current research project. The candidates were given opportunities to practice using the skills in dyads and to receive coaching and feedback from the first author and research assistants.

Training was didactic and experimental and included (1) mindfulness theory and practice (2 hours), (2) orientation to the study and role of a peer counselor (1.5 hours), ethics, confidentiality, and mandated reporting (30 minutes), (4) lunch break and personal introductions (1 hour), (5) fundamentals of counseling listening skills (30 minutes), (6) validation techniques (1.5 hours), and (7) motivational interviewing (1 hour). 

#### Peer Counselor Supervision Meetings

Weekly group supervision was attended by the research coordinator (MR), 2 research assistants, and the 4 peer counselors. Meetings were conducted in Mandarin and via a videoconferencing software (Zoom; Zoom Video Communications) after peer counselors were matched with their first participant. The structure of the supervision meetings was modeled after the elements of dialectical behavior therapy consultation team meetings [38]. The meetings began with a brief mindfulness practice and a discussion of the observations. Team members took turns leading the mindfulness practice. Next, issues were addressed according to the following hierarchy: (1) life-threatening behaviors or concerns, (2) therapy interfering behaviors, and (3) quality of life–related issues. Team members presented consultation questions and supported each other using peer counseling techniques (eg, validation and motivational interviewing), in an effort to enhance capabilities and motivation. Supervision was framed for peer counselors for both clinical consultation and peer support. The study coordinator (MR) provided 5- to 10-minute didactic lessons on the common challenges faced by peer counselors. Peer counselors also used a group chat on their mobile devices to provide each other with ongoing updates and support. They were offered the opportunity to schedule additional individual supervision from the study coordinator on an as-needed basis, or in case of a participant emergency.

### The Be Mindful Internet-Based Intervention

The *Be Mindful* course is an internet-based mindfulness training program produced by Wellmind Media, with support from the UK-based charity Mental Health Foundation. *Be Mindful* delivers all the elements of mindfulness-based cognitive therapy in an internet-based course that can be completed in 4 weeks. It can be accessed through their website [39], where its development and design are fully detailed. To date, 7 peer-reviewed papers have been published reporting study results based on the *Be Mindful* course (for full details, visit the website [40]). The course can be accessed on a computer, laptop, tablet, or smartphone. The course is self-guided, that is there is no contact with mindfulness teachers or other course participants. An overview of the course content is presented in Table 1.

When this study was conducted in May 2018, the *Be Mindful* course website reported that over 20,000 people had taken the course since 2011. The participants in this study were able to complete the course for free. Project staff received technical support and administrative access to randomize participants, track progress (eg, participant log-ins, module completion, and date of completion), and download data. All materials on the course were translated into Mandarin, including the videos, audio recordings, and homework assignments. Each week, the research coordinators emailed materials to the participants after they were ready to progress to the next chapter in the course. Furthermore, 6 weeks after initial enrollment, participants received an email thanking them for their participation and a link inviting them to complete the posttreatment assessment. If they did not complete the survey within 1 week, they were contacted via text message, WeChat, and email over the course of the next week. Participants were paid upon completion of the posttreatment questionnaire packet. 

**Table 1:** *Be Mindful* course content and assignments by week.

Chapter and title	Content	Materials and homework assignments
Before; Getting Started	Orientation to the course and format	3 videos (>6 min total); assignments: stress assessment, reflection on goals, and motivation for practicing
Week 1; Stepping out of Automatic Pilot	Introduction to the concept of mindfulness	4 videos (>12 min total); 1 audio file (30 min); assignments: events diary, body scan, routine activity, and mindful meal
Week 2; Reconnecting with Body and Breath	Awareness of thoughts and feelings	3 videos (>17 min total); 2 audio files (19 min total); assignments: difficult thoughts checklist, event awareness, mindful movement, and mindful breathing
Week 3; Working with Difficulties	Acknowledging difficult thoughts and emotions without judgment or attachment	3 videos (>9 min total); 1 audio file (22 min); assignments: stress awareness, sitting meditation, and breathing space
Week 4; Mindfulness in Daily Life	Awareness of (1) personal patterns, (2) associations to changes in mind and body, and (3) stress indicators	3 videos (>11 min total); assignments: list of four helpful and unhelpful strategies, activity awareness, breathing space, and chosen practice
After; Going Forward	Reflecting on lessons learned	3 videos (>5 min total); assignments: stress assessment, letter to yourself, and review additional resources

### Institutional Review Board Approval, Consent, and Compensation

Participants were compensated for completing the baseline questionnaire packet, posttreatment questionnaires, and for responding to each daily assessment; the total amount that the participants could make from this course was approximately US $28. This study received institutional review board approval from the Psychology Research Ethics Committee at the Beijing Institute of Technology, and all participants electronically signed a digital informed consent form.

### The MIND+ Condition

Participants randomized to the MIND+ condition (n=27) completed the same procedures as those in the MIND condition (n=27). However, those in the MIND+ condition were informed by the study coordinator via email that they were paired with a peer counselor who would provide them support and encouragement. Participants were instructed to contact their peer counselor within a week to schedule a time to chat. Peer counselors were instructed to contact their participants if they did not hear from them within 5 days. Peer counselors were encouraged to provide brief (15-20 minutes) weekly meetings to support and encourage participants in their completion of the internet-based intervention.

### Daily Reporting

Daily assessments were completed using the Qualtrics software. Participants rated their state mindfulness and mood (stress, depression, and happiness) on a 5-point Likert scale (1=*very low*, 5=*very high*). Daily questionnaires also assessed participants’ self-reported frequency and duration (in minutes) of mindfulness practice the previous day. Links to questionnaires expired within 4 hours if not completed.

### Self-report Questionnaires

A self-report questionnaire packet was completed at screening, baseline, postintervention, and at the 1-month follow-up after the end of the intervention.

The Demographic Data Survey-Modified is a self-report measure used to obtain demographic information (gender, age, university, year in school, and field of study), as well as self-report data about the patient’s English proficiency, meditation experience (previous training and current practice), psychiatric diagnostic and treatment history, and emergency contact information.

The 7-item Generalized Anxiety Disorder (GAD-7) questionnaire is a 7-item measure of the severity of anxiety symptoms in the last 2 weeks [41,42]. The Cronbach α reliability coefficient for the GAD-7 in this sample was .87.

The Patient Health Questionnaire-9 (PHQ-9) is a 9-item measure of the severity of depression symptoms in the last 2 weeks [43,44]. The Cronbach α reliability coefficient for the PHQ-9 in this sample was .85.

The Five-Factor Mindfulness Questionnaire (FFMQ), originally developed by Baer, is a 39-item measure of trait mindfulness that is organized into 5 subscales (Observing, Describing, Nonjudging of inner experience, Nonreactivity to Inner Experience, and Acting with Awareness), with 7 or 8 items in each subscale [45,46]. Only the full-scale FFMQ score was used in the analyses. The Cronbach α reliability coefficients for the total scale at baseline and posttreatment were .72 and .89, respectively.

The Depression Anxiety Stress Scale is a 21-item measure comprising 3 subscales (Depression, Anxiety, and Stress) of 7 items each, which provide indices of depression [47]. The Cronbach α reliability coefficients for depression, anxiety, and stress in this sample were .82, .74, and .77, respectively.

Perceived Stress Scale (PSS) is a 14-item measure of perceived stress in the last month [48]. The Cronbach α reliability coefficient for the PSS in this sample was .893.

### Statistical Analyses

All analyses were conducted in SAS (version 9.4, SAS Institute).

#### Coding of Time

To capture any nonlinear changes across the study, the phases were coded as follows: *early study* (days 1-11), *mid study* (days 12-23), and *late study* (days 24-35).

#### Retention

First, a chi-square analysis was used to test the hypothesis that participants randomly assigned to MIND+ (vs those assigned to MIND) would show less attrition as indicated by higher completion (vs noncompletion) rates of posttreatment assessment (yes or no).

#### Adherence

Second, independent samples, two-tailed *t* tests were used to test the hypotheses that participants randomly assigned to MIND+ (vs those assigned to MIND) would show greater program adherence, as indicated by more frequent use of the course (higher number of total log-ins) and a higher percentage of the course completed.

#### Psychosocial Outcomes

Three multilevel models (identical to those used for the number of minutes of mindfulness practice mentioned above) were used to test the hypothesis that the randomization to the MIND+ condition would result in a greater increase in mindfulness across the trial, and greater decrease in depression and stress levels across the trial. Time was also alternatively defined by examining contrasts of the beginning of the study (days 1-11) with both the middle (days 12-23) and end (days 24-35) of the study.

Model fitting was accomplished using the −2 log likelihood model to determine model fit. Random slopes were retained when this improved the model fit. Person-standardized daily values (today’s value minus overall person mean, divided by overall person SD) were used for graphical depictions of continuous outcomes to depict only the within-person changes in the outcome across the study, consistent with multilevel modeling results.

#### Effect Size

The size of group differences in each outcome, or change over time in each outcome, was estimated using Cohen *d*. For multilevel modeling outcomes, Cohen *d* was calculated for the group means of difference between scores on days in the early study phase and the late study phase.

#### Randomization

The randomization sequence was sourced through random.org [49], an automated, web-based randomization service that generates randomness using atmospheric noise. Using random.org, two 25-person blocks were used to randomize the participants into 2 equally-sized groups.

## Results

### Participant Flow and Descriptive Statistics

**Figure 1:**
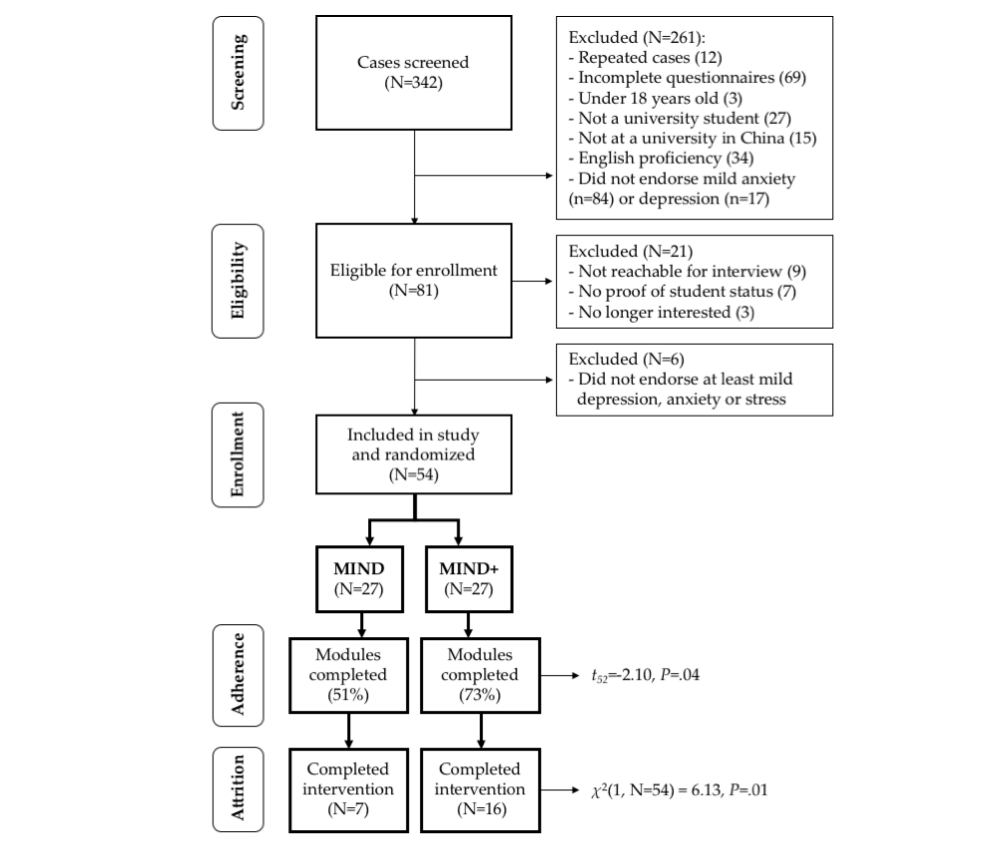
PRISMA (Preferred Reporting Items for Systematic Reviews and Meta-Analyses) flow chart.

Figure 1 shows a flow chart illustrating participant flow from recruitment to study completion. Table 2 provides descriptive statistics for demographics and key study variables for both the total sample and for each condition.

**Table 2:** Descriptive statistics in the full sample and by condition (N=54).

Variable		Total sample (N=54)	MIND (n=27)	MIND+ (n=27)	Comparisons	
Sex (female), n (%)		40 (74)	18 (67)	22 (81)	*X*^2^_1_=1.5; *P*=.21; φ^a^=0.17	
Age (years), mean (SD)		23.53 (3.17)	23.77 (3.72)	23.29 (2.56)	*t*_52_=0.55; *P=*.58; Cohen *d*=0.15	
**Mindfulness experience, n (%)**						*X*^2^_2_=3.2; *P*=.20; Cramer V=0.24
	Previously practiced	4 (7)	2 (7)	2 (7)		
	Currently practice	3 (6)	3 (11)	0		
	Never practiced	47 (87)	22 (81)	25 (93)		
**English language competence, n (%)**						*X*^2^_2_=4.6; *P*=.10; Cramer V=0.29
	CET^b^ level 4	7 (13)	6 (22)	1 (4)		
	CET level 6	38 (70)	16 (59)	22 (81)		
	TOEFL^c^ or IELTS^d^	9 (17)	5 (19)	4 (15)		
**Patient Health Questionnaire-9, mean (SD)**						
	Pretreatment	10.63 (5.0)	9.70 (4.7)	11.56 (5.3)	*t*_52_=−1.38; *P*=.17; Cohen *d=*−0.37	
	Posttreatment	7.11 (4.8)	7.42 (5.4)	6.78 (4.2)	*t*_45_=0.45; *P*=.65; Cohen *d*=0.13	
	Pre-to-post change	−3.23 (5.29)	−2.38 (5.08)	−4.13 (5.46)	*t*_45_=1.14; *P*=.26; Cohen *d*=0.33	
**7-item General Anxiety Disorder, mean (SD)**						
	Pretreatment	8.65 (4.1)	8.33 (4.0)	8.96 (4.2)	*t*_52_=−0.57; *P*=.57; Cohen *d*=−0.15	
	Posttreatment	5.96 (4.2)	5.83 (3.3)	6.09 (5.1)	*t*_45_=−0.21; *P*=.83; Cohen *d*=−0.06	
	Pre-to-post change	−2.77 (3.97)	−2.62 (3.69)	−2.91 (4.32)	*t*_45_=−0.25; *P*=.80; Cohen *d*=−0.07	
**DASS-21^e^ depression, mean (SD)**						
	Pretreatment	6.45 (3.8)	5.73 (2.8)	7.15 (4.6)	*t*_52_=−1.39; *P*=.17; Cohen *d*=−0.38	
	Posttreatment	3.96 (3.0)	3.79 (3.0)	4.13 (3.1)	*t*_45_=−0.38*; P*=.70; Cohen *d*=−0.11	
	Pre-to-post change	−2.40 (3.84)	−2.62 (3.69)	−2.87 (4.32)	*t*_45_=0.83; *P*=.41; Cohen *d*=0.24	
**DASS-21 anxiety, mean (SD)**						
	Pretreatment	6.38 (3.2)	6.19 (3.1)	6.56 (3.4)	*t*_52_=−0.42; *P*=.57; Cohen *d*=−0.11	
	Posttreatment	4.72 (2.7)	4.79 (3.0)	4.65 (2.3)	*t*_45_=0.18; *P*=.86; Cohen *d*=0.05	
	Pre-to-post change	−1.47 (3.44)	−1.29 (3.26)	−1.65 (3.22)	*t*_45_=0.36; *P*=.72; Cohen *d*=0.10	
**DASS-21 stress, mean (SD)**						
	Pretreatment	8.91 (3.4)	8.31 (3.5)	9.48 (3.3)	*t*_52_=−1.27; *P*=.21; Cohen *d*=−0.35	
	Posttreatment	6.70 (3.6)	6.33 (2.9)	7.09 (4.3)	*t*_45_=−0.71; *P*=.48; Cohen *d*=−0.21	
	Pre-to-post change	−2.17 (3.79)	−2.0 (3.78)	−2.34 (3.86)	*t*_45_=0.31; *P*=.76; Cohen *d*=0.09	
**Five-Factor Mindfulness Questionnaire, mean (SD)**						
	Pretreatment	115.46 (10.3)	116.27 (8.9)	114.65 (11.6)	*t*_52_=−0.57; *P*=.57; Cohen *d*=0.16	
	Posttreatment	119.96 (14.8)	121.63 (12.8)	118.22 (16.7)	*t*_45_=0.79; *P*=.44; Cohen *d*=0.23	
	Pre-to-post change	4.87 (13.18)	6.04 (9.84)	3.59 (16.21)	*t*_44_=0.63; *P*=.53; Cohen *d*=0.18	
Completed course, n (%)		23 (43)	7 (26)	16 (59)	*X*^2^_1_=6.1; *P*=.01; φ=0.34	
Percentage of course completion, mean (SD)		61.66 (39.37)	50.74 (37.40)	72.59 (38.88)	*t*_52_=2.10; *P*=.04; Cohen *d*=−0.57	
Number of log-ins, mean (SD)		15.72 (12.35)	13.92 (9.30)	17.51 (12.39)	*t*_52_=−1.07; *P=*.28; Cohen *d*=−0.33	

^a^φ=phi coefficient (ie, mean square contingency coefficient).

^b^CET: College English Test.

^c^TOEFL: Test of English as a Foreign Language.

^d^IELTS: International English Language Testing System.

^e^DASS-21: Depression Anxiety and Stress Scale.

### Hypotheses and Data

Hypothesis 1 predicted that participants randomly assigned to MIND+ (vs those assigned to MIND) would show less attrition, as indicated by a greater likelihood of completing the posttreatment assessment (as a dichotomous, between-person variable). A chi-square analysis comparing dichotomous condition assignment (MIND vs MIND+) and posttreatment assessment (completed vs not completed) revealed a greater number of completers in the MIND+ condition (χ^2^_1_=6.1; *P*=.01). 

Hypothesis 2 predicted that participants randomly assigned to MIND+ (vs MIND) would show greater program adherence, as indicated by a higher percentage of course completion as a continuous, between-person variable. An independent samples *t* test indicated a higher mean percentage of course completion in the MIND+ condition than in the MIND condition (mean 61.66%, SD 39.37; mean difference 21.85, 95% CI 42.69-1.010; *t*_52_=2.10; *P*=.04). 

**Figure 2:**
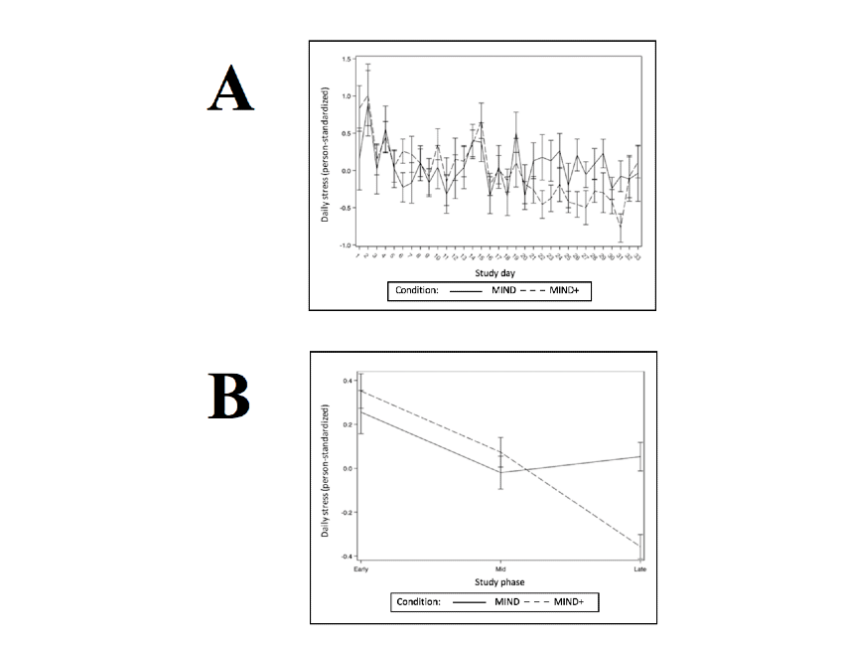
Daily (A) and phase (B) means for outcome daily self-reported stress.

**Figure 3:**
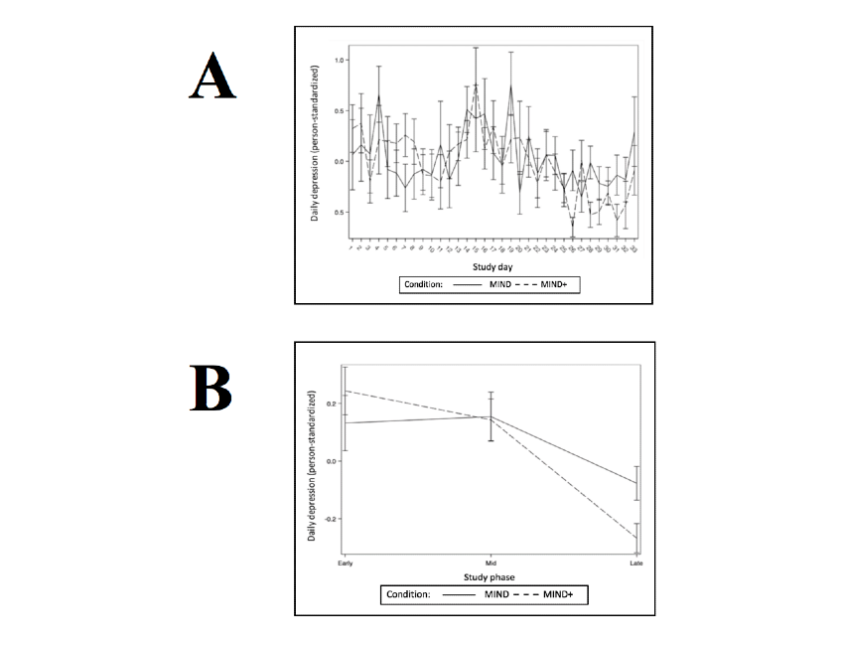
Daily (A) and phase (B) means for outcome daily self-reported depression.

**Figure 4:**
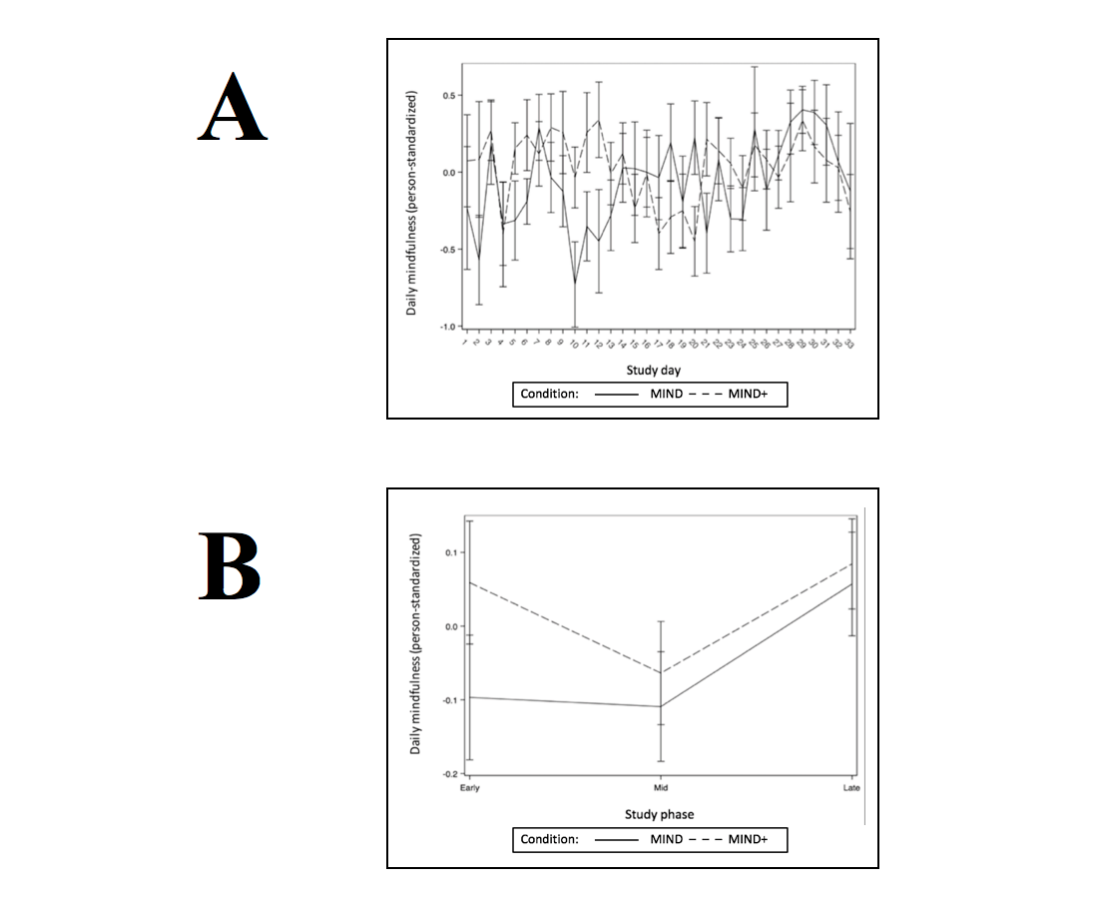
Daily (A) and phase (B) means for outcome daily self-reported mindfulness.

Hypothesis 3 predicted that participants randomly assigned to MIND+ (vs those assigned to MIND) would show more robust improvements in stress, depression, and mindfulness levels across the trial (as continuous, daily within-person variables). The results of the multilevel models testing this hypothesis are presented in Tables 3-14. Consistent with successful randomization, there were no condition effects on the baseline levels of stress, depression, or mindfulness. Both stress and depression decreased linearly as the number of study days increased, and this linear effect did not differ by condition. There were no linear effects of study days on daily mindfulness, and this effect did not differ by condition. For the daily outcomes of stress and depression, those randomized to MIND+ demonstrated a significantly greater decline from study phase 1 to phase 3. Graphs depicting daily outcomes (person-standardized) over time and phase are presented in Figures 2-4.

**Table 3:** Covariance parameters for the interactive effect of condition and time (study day) predicting daily self-reported stress.

Parameter	Estimate (SE)	Z value^a^	*P* value
Intercept	0.513 (0.192)	2.68	.004
Covariance (I,S)^b^	−0.006 (0.005)	−1.18	.24
Study day	0.000 (0.000)	2.20	.01
Residual (VC^c^)	0.556 (0.029)	19.19	<.001

^a^The Z value represents the test value of the z distribution on which statistical significance is determined for this analysis.

^b^Covariance between the random parameters for intercept and slope in the multilevel model.

^c^VC: variance component (method for structuring the covariance matrix).

**Table 4:** Fixed effects for the interactive effect of condition and time (study day) predicting daily self-reported stress.

Effect	Estimate (SE)	*t* test (*df*)	*P* value
Intercept	2.998 (0.185)	16.24 (34.1)	<.001
Condition^a^	−0.279 (0.268)	−1.04 (35.2)	.30
Study day	−0.022 (0.006)	−3.90 (39.4)	<.001
Condition X study day	0.009 (0.008)	1.08 (40.3)	.29

^a^Condition is coded as a dichotomous variable, where 0=MIND only and 1=MIND+.

**Table 5:** Covariance parameters for the interactive effect of condition and time (study phase) predicting daily self-reported stress.

Parameter	Estimate (SE)	Z value^a^	*P* value
Intercept	0.460 (0.112)	4.12	<.001
Study phase (3 vs 1)	0.340 (0.036)	9.55	<.001
Residual (VC^b^)	0.601 (0.031)	19.53	<.001

^a^The Z value represents the test value of the z distribution on which statistical significance is determined for this analysis.

^b^VC: variance component (method for structuring the covariance matrix).

**Table 6:** Fixed effects for the interactive effect of condition and time (study phase) predicting daily self-reported stress.

Effect	Estimate (SE)	*t* test (*df*)	*P* value
Intercept	2.580 (0.167)	15.48 (62.4)	<.001
Condition^a^	0.118 (0.230)	0.51 (62.2)	.61
Study phase (2 vs 1)	−0.268 (0.111)	−2.42 (360)	.02
Study phase (3 vs 1)	−0.285 (0.112)	−2.54 (272)	.01
Condition X phase (2 vs 1)	0.093 (0.151)	0.62 (344)	.54
Condition X phase (3 vs 1)	−0.234 (0.113)	−2.07 (266)	.04

^a^Condition is coded as a dichotomous variable, where 0=MIND only and 1=MIND+.

**Table 7:** Covariance parameters for the interactive effect of condition and time (study day) predicting daily self-reported depression.

Parameter	Estimate (SE)	Z value^a^	*P* value
Intercept	0.566 (0.191)	2.97	.002
Covariance (I,S)^b^	−0.007 (0.005)	−1.49	.14
Study day	0.000 (0.000)	2.19	.01
Residual (VC^c^)	0.505 (0.027)	18.92	<.001

^a^The Z value represents the test value of the z distribution on which statistical significance is determined for this analysis.

^b^Covariance between the random parameters for intercept and slope in the multilevel model.

^c^VC: variance component (method for structuring the covariance matrix).

**Table 8:** Fixed effects for the interactive effect of condition and time (study day) predicting daily self-reported depression.

Effect	Estimate (SE)	*t* test (*df*)	*P* value
Intercept	2.439 (0.189)	12.94 (38.1)	<.001
Condition^a^	−0.160 (0.273)	−0.59 (39.1)	.56
Study day	−0.013 (0.005)	−2.40 (39.2)	.02
Condition X study day	0.008 (0.008)	1.01 (40.1)	.32

^a^Condition is coded as a dichotomous variable, where 0=MIND only and 1=MIND+.

**Table 9:** Covariance parameters for the interactive effect of condition and time (study phase) predicting daily self-reported depression.

Parameter	Estimate (SE)	Z value^a^	*P* value
Intercept	0.422 (0.104)	4.08	<.001
Study phase (3 vs 1)	0.359 (0.035)	10.29	<.001
Residual (VC^b^)	0.544 (0.028)	19.30	<.001

^a^The Z value represents the test value of the z distribution on which statistical significance is determined for this analysis.

^b^VC: variance component (method for structuring the covariance matrix).

**Table 10:** Fixed effects for the interactive effect of condition and time (study phase) predicting daily self-reported depression.

Effect	Estimate (SE)	*t* test (*df*)	*P* value
Intercept	2.164 (0.160)	13.55 (61.5)	<.001
Condition^a^	0.117 (0.221)	0.53 (61.3)	.60
Study phase (2 vs 1)	0.025 (0.106)	0.23 (362)	.82
Study phase (3 vs 1)	−0.063 (0.108)	−0.58 (271)	.56
Condition X phase (2 vs 1)	−0.162 (0.145)	−1.12 (347)	.26
Condition X phase (3 vs 1)	−0.280 (0.128)	−2.19 (265)	.03

^a^Condition is coded as a dichotomous variable, where 0=MIND only and 1=MIND+.

**Table 11:** Covariance parameters for the interactive effect of condition and time (study day) predicting daily self-reported mindfulness.

Parameter	Estimate (SE)	Z value^a^	*P* value
Intercept	0.395 (0.134)	2.95	.002
Covariance (I,S)^b^	−0.006 (0.004)	−1.64	.10
Study day	0.000 (0.000)	2.49	.006
Residual (VC^c^)	0.526 (0.025)	21.09	<.001

^a^The Z value represents the test value of the z distribution on which statistical significance is determined for this analysis.

^b^Covariance between the random parameters for intercept and slope in the multilevel model.

^c^VC: variance component (method for structuring the covariance matrix).

**Table 12:** Fixed effects for the interactive effect of condition and time (study day) predicting daily self-reported mindfulness.

Effect	Estimate (SE)	*t* test (*df*)	*P* value
Intercept	2.561 (0.164)	15.61 (40.5)	<.001
Condition^a^	0.007 (0.237)	0.03 (42.0)	.98
Study day	0.002 (0.005)	0.30 (41.2)	.76
Condition X study day	0.007 (0.008)	0.93 (42.1)	.36

^a^Condition is coded as a dichotomous variable, where 0=MIND only and 1=MIND+.

**Table 13:** Covariance parameters for the interactive effect of condition and time (study phase) predicting daily self-reported mindfulness.

Parameter	Estimate (SE)	Z value^a^	*P* value
Intercept	0.300 (0.073)	4.13	<.001
Study phase (3 vs 1)	0.211 (0.037)	5.75	<.001
Residual (VC^b^)	0.565 (0.027)	21.26	<.001

^a^The Z value represents the test value of the z distribution on which statistical significance is determined for this analysis.

^b^VC: variance component (method for structuring the covariance matrix).

**Table 14:** Fixed effects for the interactive effect of condition and time (study phase) predicting daily self-reported mindfulness.

Effect	Estimate (SE)	*t* test (*df*)	*P* value
Intercept	2.732 (0.139)	19.69 (69.6)	<.001
Condition^a^	−0.142 (0.191)	−0.74 (69.3)	.46
Study phase (2 vs 1)	0.023 (0.100)	0.23 (405.0)	.82
Study phase (3 vs 1)	0.123 (0.099)	1.24 (324.0)	.22
Condition X phase (2 vs 1)	−0.084 (0.136)	−0.62 (383.0)	.54
Condition X phase (3 vs 1)	−0.053 (0.136)	−0.39 (314.0)	.69

^a^Condition is coded as a dichotomous variable, where 0=MIND only and 1=MIND+.

## Discussion

### Principal Findings

The aim of this study was to investigate the efficacy of an adjunctive treatment component that uses task-shifting (ie, nonspecialist peer counselors) to enhance engagement in a self-directed, web-based mindfulness intervention for stress and depression among Chinese undergraduate and graduate students.

The results indicated that participants assigned to the MIND+ (vs those assigned to the MIND) condition showed significantly less attrition and more adherence, as indicated by a greater likelihood of completing posttreatment assessments and a higher percentage of course completion, respectively. In addition, individuals in the MIND+ condition reported significant improvements in daily ratings of stress and depression levels across the trial compared with individuals in the MIND condition. These findings suggest that volunteer peer counselors receiving brief training and weekly supervision may significantly improve participants’ indices of treatment engagement and mental health outcomes in an internet-based mindfulness intervention among college and graduate students in China.

### Unique Contributions of This Study

This study makes several unique contributions to the literature. First, an internet-based platform was used to deliver a mindfulness intervention in a sample of individuals from a non-Western LMIC. There have been no publications of results from randomized controlled trials investigating the efficacy of a self-guided, web-based, mindfulness intervention in China.

Second, this study uses a task-shifting informed approach aimed at increasing retention and adherence to an existing evidence-based intervention. There have been no publications of results from randomized controlled trials investigating the efficacy of an adjunctive, web-based, peer support intervention component intended to enhance treatment engagement in a self-guided, web-based, mental health intervention in an LMIC. Furthermore, only 3 studies have been published on task shifting in mental health services of any type in China [50-52]. Accordingly, the results of this study advance the literature in this area, providing promise in the use of task shifting to improve mental health outcomes in China, the world’s most populous country.

Third, this study explored the effects of a very low-intensity, low-cost task-shifting intervention. Nonspecialist peer counselors received only 1 day of in-person training with ongoing web-based, group supervision once per week. Furthermore, each participant in the MIND+ group only received a mean of 4.69 peer counseling *sessions* (SD 2.04; range 0-7), lasting an average total of 120.85 minutes (SD 66.53; range 12-345) per MIND+ participant. All peer counselors maintained full-time student status *and* part-time jobs throughout the course of volunteering for this study. Peer counselors reported completion of responsibilities for this study (including supervision, peer counseling, and documentation) and required, on average, less than 2 hours per week, when supporting 2 patients at the same time.

Fourth, this study contributes new insights into the selection, supervision, and evaluation practices for task-shifting initiatives. According to a recent systematic review of 137 studies from 48 countries employing task shifting to deliver evidence-based mental health services in LMICs, fewer than 1 in 5 studies reported providing supervision on a weekly or biweekly basis [53]. In this study, nonspecialist peer counselors were evaluated for knowledge (questionnaires), application (group supervision discussions), competence (role-plays during training and in group supervision), and quality of care (including patients’ evaluations, pre to postsession changes in participants’ self-reported mood and motivation, and 2 *session* audio recordings). 

### Completion Rates in This Study Versus Those in Comparators

Completion rates in this study (7/27, 26% for MIND and 16/27, 59% for MIND+) appear to be lower than those in previous studies. In comparison, Querstret et al [54] reported 73.7% (87/118) of participants completing the web-based program. Krusche et al [55] reported 29.39% (1497/5094) of participants completing the course *and* follow-up assessments; however, only 11% (3/27) of MIND participants and 15% (4/27) of MIND+ participants completed both.

One explanation for the lower completion rates in this study is that participants completed the course in their second language. Of the 43 participants who completed the posttreatment assessment, when asked the degree to which language was a barrier in completing the course, 16 (37%) indicated *not at all*, and 17 (40%) reported *a little*. However, 9% (4/43) of participants indicated that language was *very much* a barrier to completion. It is also possible that language was more of a barrier for the 11 participants who did not complete the posttreatment assessment. However, follow-up analyses did not reveal an association between language proficiency and course completion or engagement. Specifically, follow-up analyses revealed that self-reported baseline English proficiency and posttreatment perception of language as a barrier to completion were not significant predictors of treatment completion.

Another explanation for this difference is that participants in the studies by Krusche et al [55] and Querset et al [54] were paying US $60-$90 for participation; therefore, they were perhaps more motivated. Before the start of the study, 26% (14/54) participants reported being *very* motivated to learn and practice mindfulness, and 44% (24/54) reported being *extremely* motivated. However, across both groups, baseline self-reported energy to learn and practice mindfulness was predictive of failure to activate the *Be Mindful* course (*r*=0.288; *P*=.04) and total login count (*r*=0.300; *P*=.03).

Participants assigned to the MIND+ (vs those assigned to the MIND) condition showed significantly less attrition, as indicated by a greater likelihood of completing the posttreatment assessments in the internet-based course. MIND+ participants also demonstrated a nonsignificant trend toward lower rates of nonuse attrition (*P*=.05), defined as never responding to the daily assessment or not responding for at least the last 3 weeks of the study. These findings suggest that a low-cost, low-intensity task-shifting component is promising as a feasible and scalable approach for enhancing retention in web-based evidence-based treatments. Previous research suggests that personalizing the contact (eg, provision of therapist name and photo vs a virtual therapist or personalized vs standardized messaging) is associated with lower rates of treatment termination [28,56]. Apart from strategies that involve human support, future research can also continue to explore automated strategies to provide more personalized treatment experience in self-guided, internet-based mindfulness interventions.

### Unique Findings of This Study

Participants assigned to the MIND+ (vs those assigned to MIND) condition showed greater program adherence, as indicated by a higher percentage of the course completed. However, there were no between-group differences in attrition, as indicated by (1) more frequent log-ins to the course, (2) a less robust decrease in daily self-reports of mindfulness practice, or (3) a less robust decrease in daily self-reports of minutes of mindfulness practiced over the course of the treatment. Overall, these data suggest that the MIND+ task-shifting component increased participants’ likelihood of completing the program but not necessarily their likelihood to be more actively engaged in the program (ie, more frequent log-ins) or to report higher frequency or duration of mindfulness practice.

It is worth noting that participants in this study presented with mean baseline PSS (stress), GAD-7 (anxiety), and PHQ-9 (depression) scores of 23.27 (SD 4.28), 9.90 (SD 3.98), and 11.31 (SD 5.06), respectively. These means are higher than the scores provided in published population norms for the PSS (between 11.9 and 14.7) [57], GAD-7 (between 2.7 and 3.8) [58,59], and PHQ-9 (approximately 3.3) [42]. Instead, the participants in this study would, on average, be considered *highly stressed* [59,60], *moderately anxious* [41,61], and *moderately depressed* [42].

Although participants were randomized to study conditions, the MIND+ group participants reported significantly higher mean baseline PSS scores than those of the MIND group participants. PSS was the only baseline measure with significant between-group differences in this study. However, it is possible that this difference in stress helps in explaining why MIND+ participants completed more modules but did not report more frequent and longer-lasting mindfulness practice than MIND participants. On the other hand, it is possible that MIND+ participants reported more stress because they were assigned to the condition with a peer counselor, and they felt more pressure to complete the course. Existing research suggests that positive, high-quality social support can enhance resilience to stress and reduce depressive symptomology and medical morbidity and mortality [62,63]. Nevertheless, future research could explore the mechanisms by which peer support enhances adherence, including factors such as social desirability, expectation, and compliance.

Enrollment in this study was associated with a significant increase in reported trait mindfulness, as indicated by the FFMQ scores. This was true across both groups, with no significant between-group differences. Further analyses should explore whether changes in mindfulness mediate the effects of interventions on depression, anxiety, and stress. The results of Querstret et al [54] showed that although the intervention worked to increase levels of the *describing* and *nonjudging* facets of mindfulness, only Acting with Awareness mediated the effects of the intervention on mental health outcomes.

This study improves upon previous studies of daily practice in relation to the *Be Mindful* internet-based course. Krusche et al [55,64] asked participants to rate their mindfulness activities once per week using a high (*every day or most days*), medium (*sometimes*), and low (*rarely or never*) scale [64]. In the 2013 study, 51.65% (141/273) of participants reported practicing *sometimes*, and in the 2012 study, 55% (55/100) of participants reported practicing *sometimes*. This study improves upon this approach by collecting daily data and having participants record the number of minutes practiced per day. However, future research would benefit from collecting separate data for formal and informal mindfulness practice, or by collecting data in real time (eg, asking participants to report immediately before and after mindfulness practice).

### Significance of These Findings

The results of this study indicate that participation in the internet-based intervention was associated with significant improvements in pre to posttreatment stress outcomes. The pre-post effect size (Cohen *d*) for stress among the completers was 1.13. This was equivalent to those reported in previous studies of the *Be Mindful* course [55], web-based interventions [65,66], and in-person mindfulness interventions [17,67,68].

These findings are significant because stress has been shown to be associated with a wide range of physical and mental health problems [69,70], including autoimmune diseases, depression, substance abuse, and suicidal behavior. Research suggests that the prevalence of stress among college students, particularly Chinese students [71], is increasing [72]. Students from Confucian Asian countries (eg, Japan, Korea, and China) report higher levels of stress, anxiety, and self-doubt than students from European regions [73]. Moreover, compared with their Korean and Japanese counterparts, Chinese college students reported the highest number of stressors and the highest levels of stress, along with passive and ineffective coping [74,75].

The pre-post effect sizes (Cohen *d*) for anxiety (GAD-7) and depression (PHQ-9) among completers were 0.89 and 0.95, respectively, which are equivalent to those reported in previous studies of the *Be Mindful* course and in-person mindfulness interventions [17,55,66].

In addition, individuals in the MIND+ condition reported significant improvements in daily ratings of stress and depression across the trial compared with individuals in the MIND condition. These findings suggest that volunteer peer counselors receiving brief training and weekly supervision may significantly improve participants’ indices of treatment engagement and mental health outcomes in an internet-based mindfulness intervention among college and graduate students in China. It is worth noting that these differences between groups were not linear across the course of the study. The benefits of assignment to the MIND+ group appear late during the treatment, that is, between phases 2 and 3. In the middle of the study, MIND+ participants did not report less anxiety or depression, and they did not report practicing more than the MIND-only participants. Therefore, one explanation for the benefit of the program was weekly contact with peer counselors. Another explanation is that they received more content during the intervention. The effect size of the internet-based course on stress, depression, and anxiety scores suggests that this treatment is effective for Chinese students, regardless of whether they have contact with peers or a therapist.

MIND+ participants did not report significant improvements in daily ratings of state mindfulness across the trial compared with participants in the MIND-only condition. Instead, there was a main effect of treatment on improvements on daily mindfulness ratings. Similarly, there were no between-group differences in pre-post FFMQ scores, although there was a moderate main effect of mindfulness (FFMQ) among completers (Cohen *d*=0.55). These outcomes suggest that the effect of having peer support did not increase reported mindfulness as measured in daily assessments or in pre-post measures. Furthermore, it is unlikely that the effect of practice and change in trait mindfulness did not mediate the change in daily reports of stress and depression over the course of the treatment.

### Study Limitations

This study has several limitations. First, the sample was small, achieving 80% power to detect only moderately-sized group differences (ie, Cohen *d*=0.78 or larger). The sample also consisted of a nonclinical sample of English-speaking university students. Before these findings can be generalized, this research should be replicated among participants using the internet-based intervention in their native language and among larger, more diverse samples. Related to this, the significant effects described here would not survive correction for the number of tests performed in this study; nonetheless, we believe that it is important to share our findings with the field. Second, there were insufficient follow-up data to be able to analyze whether the effects of treatment were sustained over time. Data related to peer counselors’ communication with participants were not included in these analyses. It is possible that the frequency and duration of peer counseling influences the effect of treatment conditions on engagement and mental health outcomes. Future studies should explore and detect the possible dosage effects. Moreover, future research would benefit from having more objective indicators of study, practice, and mindfulness meditation. For example, one patient might report practicing mindfulness once a day, but it could be a 45-minute body scan, and another could report practicing 35 times because they could have noticed their thoughts or body sensations that many times throughout the day. Finally, the exclusion of candidates who expressed suicidal ideation might have led to an underestimation of the overall treatment effect, because the cohort most severely affected by depression was not considered in the study.

### Conclusions

This study provides preliminary support for the effectiveness of a 4-week, internet-based mindfulness course for the reduction of self-reported symptoms of stress, depression, and anxiety among English-speaking university students in China. The effects were compared with those reported in other mindfulness courses delivered on the web and in-person. Furthermore, these results highlight the potential of leveraging task shifting to enhance treatment engagement in self-guided evidence-based treatments. The combination of these approaches may represent a financially feasible, easily transportable, and quickly scalable way to provide mental health services in low-resource settings.

## Appendix

Multimedia Appendix 1 CONSORT-EHEALTH checklist (V 1.6.1).

## Abbreviations

CET: College English Test
FFMQ: Five-Factor Mindfulness Questionnaire
GAD-7: 7-item Generalized Anxiety Disorder
LMIC: low- and middle-income country
PHQ-9: Patient Health Questionnaire-9
PSS: Perceived Stress Scale
