# Supplementary material for: The Use of Task Shifting to Improve Treatment Engagement in an Internet-Based Mindfulness Intervention Among Chinese University Students: Randomized Controlled Trial
Source: JMIR Form Res. 2021 Oct 13;5(10):e25772. doi: 10.2196/25772 (PMC8552103; doi:10.2196/25772)
Supplement: Multimedia Appendix 1 [file formative_v5i10e25772_app1.pdf]

CONSORT-EHEALTH (V 1.6.1) - Submission/Publication Form

The CONSORT-EHEALTH checklist is intended for authors of randomized trials evaluating web-based and Internet-based applications/interventions, including mobile interventions, electronic games (incl multiplayer games), social media, certain telehealth applications, and other interactive and/or networked electronic applications. Some of the items (e.g. all subitems under item 5 - description of the intervention) may also be applicable for other study designs.

The goal of the CONSORT EHEALTH checklist and guideline is to be
a) a guide for reporting for authors of RCTs,
b) to form a basis for appraisal of an ehealth trial (in terms of validity)

CONSORT-EHEALTH items/subitems are MANDATORY reporting items for studies published in the Journal of Medical Internet Research and other journals / scientific societies endorsing the checklist.

Items numbered 1., 2., 3., 4a., 4b etc are original CONSORT or CONSORT-NPT (non-pharmacologic treatment) items.
Items with Roman numerals (i., ii, iii, iv etc.) are CONSORT-EHEALTH extensions/clarifications.

As the CONSORT-EHEALTH checklist is still considered in a formative stage, we would ask that you also RATE ON A SCALE OF 1-5 how important/useful you feel each item is FOR THE PURPOSE OF THE CHECKLIST and reporting guideline (optional).

Mandatory reporting items are marked with a red *.
In the textboxes, either copy & paste the relevant sections from your manuscript into this form - please include any quotes from your manuscript in QUOTATION MARKS,
or answer directly by providing additional information not in the manuscript, or elaborating on why the item was not relevant for this study.

YOUR ANSWERS WILL BE PUBLISHED AS A SUPPLEMENTARY FILE TO YOUR PUBLICATION IN JMIR AND ARE CONSIDERED PART OF YOUR PUBLICATION (IF ACCEPTED).
Please fill in these questions diligently. Information will not be copyedited, so please use proper spelling and grammar, use correct capitalization, and avoid abbreviations.

DO NOT FORGET TO SAVE AS PDF _AND_ CLICK THE SUBMIT BUTTON SO YOUR ANSWERS ARE IN OUR DATABASE !!!

Citation Suggestion (if you append the pdf as Appendix we suggest to cite this paper in the caption):
Eysenbach G, CONSORT-EHEALTH Group
CONSORT-EHEALTH: Improving and Standardizing Evaluation Reports of Web-based and

Mobile Health Interventions
J Med Internet Res 2011;13(4):e126
URL: http://www.jmir.org/2011/4/e126/
doi: 10.2196/jmir.1923
PMID: 22209829

* Required

Your name *

First Last

Jared Weisman, B.A.

Primary Affiliation (short), City, Country *

University of Toronto, Toronto, Canada

Pitzer College, Claremont, CA, USA

Your e-mail address *

abc@gmail.com

jweisman@pitzer.edu

Title of your manuscript *

Provide the (draft) title of your manuscript.

The Use of Task-Shifting to Improve Treatment Engagement in an Internet-Based Mindfulness Intervention among Chinese University Students: A Randomized Controlled Trial

Name of your App/Software/Intervention *

If there is a short and a long/alternate name, write the short name first and add the long name in brackets.

Be Mindful (bemindfulonline.com)

Evaluated Version (if any)

e.g. "V1", "Release 2017-03-01", "Version 2.0.27913"

Your answer

Language(s) *

What language is the intervention/app in? If multiple languages are available, separate by comma (e.g. "English, French")

The app is published in English and was transl

URL of your Intervention Website or App

e.g. a direct link to the mobile app on app in appstore (itunes, Google Play), or URL of the website. If the intervention is a DVD or hardware, you can also link to an Amazon page.

https://www.bemindfulonline.com/

URL of an image/screenshot (optional)

Your answer

Accessibility *

Can an enduser access the intervention presently?

- ○ access is free and open
- ○ access only for special usergroups, not open
- ◉ access is open to everyone, but requires payment/subscription/in-app purchases
- ○ app/intervention no longer accessible
- ○ Other:

Primary Medical Indication/Disease/Condition *

e.g. "Stress", "Diabetes", or define the target group in brackets after the condition, e.g. "Autism (Parents of children with)", "Alzheimers (Informal Caregivers of)"

Stress, anxiety, and depression.

Primary Outcomes measured in trial *

comma-separated list of primary outcomes reported in the trial

Self-reported levels of stress, anxiety, depressi

Secondary/other outcomes

Are there any other outcomes the intervention is expected to affect?

Your answer

Recommended "Dose" *

What do the instructions for users say on how often the app should be used?

- (x) Approximately Daily
- ( ) Approximately Weekly
- ( ) Approximately Monthly
- ( ) Approximately Yearly
- ( ) "as needed"
- ( ) Other:

Approx. Percentage of Users (starters) still using the app as recommended after 3 months *

- ( ) unknown / not evaluated
- ( ) 0-10%
- ( ) 11-20%
- ( ) 21-30%
- ( ) 31-40%
- ( ) 41-50%
- ( ) 51-60%
- ( ) 61-70%
- ( ) 71%-80%
- ( ) 81-90%
- ( ) 91-100%
- (x) Other: The program is designed to be completed in 4 weeks.

Overall, was the app/intervention effective? *

- ○ yes: all primary outcomes were significantly better in intervention group vs control
- ○ partly: SOME primary outcomes were significantly better in intervention group vs control
- ○ no statistically significant difference between control and intervention
- ○ potentially harmful: control was significantly better than intervention in one or more outcomes
- ○ inconclusive: more research is needed
- ● Other: For both conditions, participation in the online intervention was assoc

Article Preparation Status/Stage *

At which stage in your article preparation are you currently (at the time you fill in this form)

- ○ not submitted yet - in early draft status
- ○ not submitted yet - in late draft status, just before submission
- ○ submitted to a journal but not reviewed yet
- ● submitted to a journal and after receiving initial reviewer comments
- ○ submitted to a journal and accepted, but not published yet
- ○ published
- ○ Other:

Journal *

If you already know where you will submit this paper (or if it is already submitted), please provide the journal name (if it is not JMIR, provide the journal name under "other")

- ○ not submitted yet / unclear where I will submit this
- ◉ Journal of Medical Internet Research (JMIR)
- ○ JMIR mHealth and UHealth
- ○ JMIR Serious Games
- ○ JMIR Mental Health
- ○ JMIR Public Health
- ○ JMIR Formative Research
- ○ Other JMIR sister journal
- ○ Other:

Is this a full powered effectiveness trial or a pilot/feasibility trial? *

- ○ Pilot/feasibility
- ◉ Fully powered

Manuscript tracking number *

If this is a JMIR submission, please provide the manuscript tracking number under "other" (The ms tracking number can be found in the submission acknowledgement email, or when you login as author in JMIR. If the paper is already published in JMIR, then the ms tracking number is the four-digit number at the end of the DOI, to be found at the bottom of each published article in JMIR)

- ○ no ms number (yet) / not (yet) submitted to / published in JMIR
- ◉ Other: 25772

TITLE AND ABSTRACT

1a) TITLE: Identification as a randomized trial in the title

1a) Does your paper address CONSORT item 1a? *

I.e does the title contain the phrase "Randomized Controlled Trial"? (if not, explain the reason under "other")

- (x) yes
- ( ) Other:

1a-i) Identify the mode of delivery in the title

Identify the mode of delivery. Preferably use “web-based” and/or “mobile” and/or “electronic game” in the title. Avoid ambiguous terms like “online”, “virtual”, “interactive”. Use “Internet-based” only if Intervention includes non-web-based Internet components (e.g. email), use “computer-based” or “electronic” only if offline products are used. Use “virtual” only in the context of “virtual reality” (3-D worlds). Use “online” only in the context of “online support groups”. Complement or substitute product names with broader terms for the class of products (such as “mobile” or “smart phone” instead of “iphone”), especially if the application runs on different platforms.

	1	2	3	4	5	
subitem not at all important	( )	( )	( )	(x)	( )	essential

Clear selection

Does your paper address subitem 1a-i? *

Copy and paste relevant sections from manuscript title (include quotes in quotation marks "like this" to indicate direct quotes from your manuscript), or elaborate on this item by providing additional information not in the ms, or briefly explain why the item is not applicable/relevant for your study

"...An Internet-Based Mindfulness Intervention..."

1a-ii) Non-web-based components or important co-interventions in title

Mention non-web-based components or important co-interventions in title, if any (e.g., “with telephone support”).

	1	2	3	4	5	
subitem not at all important	○	○	○	○	○	essential

Does your paper address subitem 1a-ii?

Copy and paste relevant sections from manuscript title (include quotes in quotation marks "like this" to indicate direct quotes from your manuscript), or elaborate on this item by providing additional information not in the ms, or briefly explain why the item is not applicable/relevant for your study

"The Use of Task-Shifting to Improve Treatment..."

1a-iii) Primary condition or target group in the title

Mention primary condition or target group in the title, if any (e.g., “for children with Type I Diabetes”)
Example: A Web-based and Mobile Intervention with Telephone Support for Children with Type I Diabetes: Randomized Controlled Trial

	1	2	3	4	5	
subitem not at all important	○	○	○	◉	○	essential

Clear selection

Does your paper address subitem 1a-iii? *

Copy and paste relevant sections from manuscript title (include quotes in quotation marks "like this" to indicate direct quotes from your manuscript), or elaborate on this item by providing additional information not in the ms, or briefly explain why the item is not applicable/relevant for your study

"...among Chinese University Students..."

1b) ABSTRACT: Structured summary of trial design, methods, results, and conclusions

NPT extension: Description of experimental treatment, comparator, care providers, centers, and blinding status.

1b-i) Key features/functionalities/components of the intervention and comparator in the METHODS section of the ABSTRACT

Mention key features/functionalities/components of the intervention and comparator in the abstract. If possible, also mention theories and principles used for designing the site. Keep in mind the needs of systematic reviewers and indexers by including important synonyms. (Note: Only report in the abstract what the main paper is reporting. If this information is missing from the main body of text, consider adding it)

	1	2	3	4	5	
subitem not at all important	○	○	◉	○	○	essential

Clear selection

Does your paper address subitem 1b-i? *

Copy and paste relevant sections from the manuscript abstract (include quotes in quotation marks "like this" to indicate direct quotes from your manuscript), or elaborate on this item by providing additional information not in the ms, or briefly explain why the item is not applicable/relevant for your study

Fifty-four students from 36 universities across China reporting at least mild stress, anxiety, and/or depression were randomly assigned to a brief, 4-week internet-based mindfulness intervention (MIND) or to the intervention plus peer counselor support (MIND+). “The “Be Mindful” internet-based course delivers all the elements of Mindfulness-Based Cognitive Therapy (MBCT) in an internet-based course that can be completed in 4 weeks.”

1b-ii) Level of human involvement in the METHODS section of the ABSTRACT

Clarify the level of human involvement in the abstract, e.g., use phrases like “fully automated” vs. “therapist/nurse/care provider/physician-assisted” (mention number and expertise of providers involved, if any). (Note: Only report in the abstract what the main paper is reporting. If this information is missing from the main body of text, consider adding it)

	1	2	3	4	5	
subitem not at all important	○	○	◉	○	○	essential

Clear selection

Does your paper address subitem 1b-ii?

Copy and paste relevant sections from the manuscript abstract (include quotes in quotation marks "like this" to indicate direct quotes from your manuscript), or elaborate on this item by providing additional information not in the ms, or briefly explain why the item is not applicable/relevant for your study

Participants in the MIND group were responsible for completing the internet-based intervention in a self-guided manner, whereas the MIND+ group received “the intervention plus peer counselor support.”

1b-iii) Open vs. closed, web-based (self-assessment) vs. face-to-face assessments in the METHODS section of the ABSTRACT

Mention how participants were recruited (online vs. offline), e.g., from an open access website or from a clinic or a closed online user group (closed usergroup trial), and clarify if this was a purely web-based trial, or there were face-to-face components (as part of the intervention or for assessment). Clearly say if outcomes were self-assessed through questionnaires (as common in web-based trials). Note: In traditional offline trials, an open trial (open-label trial) is a type of clinical trial in which both the researchers and participants know which treatment is being administered. To avoid confusion, use “blinded” or “unblinded” to indicated the level of blinding instead of “open”, as “open” in web-based trials usually refers to “open access” (i.e. participants can self-enrol). (Note: Only report in the abstract what the main paper is reporting. If this information is missing from the main body of text, consider adding it)

	1	2	3	4	5	
subitem not at all important	○	○	◉	○	○	essential

Clear selection

Does your paper address subitem 1b-iii?

Copy and paste relevant sections from the manuscript abstract (include quotes in quotation marks "like this" to indicate direct quotes from your manuscript), or elaborate on this item by providing additional information not in the ms, or briefly explain why the item is not applicable/relevant for your study

“Participants completed daily monitoring of mindfulness practice and mood, as well as baseline and post-treatment self-reported levels of depression, anxiety, stress, and trait mindfulness.” All aspects of the intervention and assessment were internet-based. Info on recruitment is provided in the methods but not the abstract: “Participants were recruited via WeChat blogs, student club listservs, and university websites listing available jobs and research opportunities. Interested individuals completed an online screening assessment.”

1b-iv) RESULTS section in abstract must contain use data

Report number of participants enrolled/assessed in each group, the use/uptake of the intervention (e.g., attrition/adherence metrics, use over time, number of logins etc.), in addition to primary/secondary outcomes. (Note: Only report in the abstract what the main paper is reporting. If this information is missing from the main body of text, consider adding it)

	1	2	3	4	5	
subitem not at all important	○	○	●	○	○	essential

Clear selection

Does your paper address subitem 1b-iv?

Copy and paste relevant sections from the manuscript abstract (include quotes in quotation marks "like this" to indicate direct quotes from your manuscript), or elaborate on this item by providing additional information not in the ms, or briefly explain why the item is not applicable/relevant for your study

“For both conditions, participation in the intervention was associated with significant improvements in mindfulness and mental health outcomes. Pre-post effect sizes (d) for mindfulness, depression, anxiety, and stress, were 0.55, 0.95, 0.89 and 1.13, respectively. MIND+ participants showed significantly less attrition and more adherence (16/27, 59% vs. 7/27, 26%; $\chi 21=6.1$, P=.013) and a greater percentage of course completion (73% vs 51%; t52=2.10, P=.040). There were no significant between-group differences in daily self-reports of frequency and duration of mindfulness practice. Multilevel logistic growth models showed that MIND+ participates reported significantly greater pre-post improvements in daily ratings of stress (interaction estimate .39, SE .18; t317=2.29, P=.022) and depression (interaction estimate .38, SE .16; t330=2.37, P=.018).”

1b-v) CONCLUSIONS/DISCUSSION in abstract for negative trials

Conclusions/Discussions in abstract for negative trials: Discuss the primary outcome - if the trial is negative (primary outcome not changed), and the intervention was not used, discuss whether negative results are attributable to lack of uptake and discuss reasons. (Note: Only report in the abstract what the main paper is reporting. If this information is missing from the main body of text, consider adding it)

	1	2	3	4	5	
subitem not at all important	○	◉	○	○	○	essential

Clear selection

Does your paper address subitem 1b-v?

Copy and paste relevant sections from the manuscript abstract (include quotes in quotation marks "like this" to indicate direct quotes from your manuscript), or elaborate on this item by providing additional information not in the ms, or briefly explain why the item is not applicable/relevant for your study

N/A – this trial did not yield negative results.

INTRODUCTION

2a) In INTRODUCTION: Scientific background and explanation of rationale

2a-i) Problem and the type of system/solution

Describe the problem and the type of system/solution that is object of the study: intended as stand-alone intervention vs. incorporated in broader health care program? Intended for a particular patient population? Goals of the intervention, e.g., being more cost-effective to other interventions, replace or complement other solutions? (Note: Details about the intervention are provided in "Methods" under 5)

	1	2	3	4	5	
subitem not at all important	○	○	○	○	●	essential

Clear selection

Does your paper address subitem 2a-i? *

Copy and paste relevant sections from the manuscript (include quotes in quotation marks "like this" to indicate direct quotes from your manuscript), or elaborate on this item by providing additional information not in the ms, or briefly explain why the item is not applicable/relevant for your study

"Traditional therapy models are incapable of closing the mental health gap in LMICs. Online/computer-based interventions are a promising solution to closing the gap in treatment. While tech-based mindfulness interventions can be effective, treatment engagement is a key barrier to implementation. High attrition and low adherence are often observed in research & practice. Past research indicates that therapist support has a positive effect on adherence and enhances the effectiveness of web-based interventions. While offering guidance may improve adherence and treatment outcomes, it is costly and may restrict scalability. Task-sharing has recently been investigated as a promising strategy to overcome human resource shortages in LMICs. In this model of care, peer counselors receive training, supervision, and oversight from mental healthcare professionals."

2a-ii) Scientific background, rationale: What is known about the (type of) system

Scientific background, rationale: What is known about the (type of) system that is the object of the study (be sure to discuss the use of similar systems for other conditions/diagnoses, if appropiate), motivation for the study, i.e. what are the reasons for and what is the context for this specific study, from which stakeholder viewpoint is the study performed, potential impact of findings [2]. Briefly justify the choice of the comparator.

	1	2	3	4	5	
subitem not at all important	○	○	○	○	◉	essential

Clear selection

Does your paper address subitem 2a-ii? *

Copy and paste relevant sections from the manuscript (include quotes in quotation marks "like this" to indicate direct quotes from your manuscript), or elaborate on this item by providing additional information not in the ms, or briefly explain why the item is not applicable/relevant for your study

"Systematic reviews and meta-analyses have demonstrated mindfulness and acceptance to be beneficial in treating physical health conditions as well as mental health problems. A recent meta-analysis explored the efficacy of mindfulness-based interventions delivered through the internet. Overall pre-post, between-group effects (Hedge's g) were reported for stress (g=0.51), depression (g=0.29), anxiety (g=0.22), well-being (g=0.23), and mindfulness (g=0.32), all with nominal statistical significance thresholds of P<0.5. Taken together, the results from this meta-analysis provide promising initial evidence for the efficacy of mindfulness-based interventions delivered through technology."

2b) In INTRODUCTION: Specific objectives or hypotheses

Does your paper address CONSORT subitem 2b? *

Copy and paste relevant sections from the manuscript (include quotes in quotation marks "like this" to indicate direct quotes from your manuscript), or elaborate on this item by providing additional information not in the ms, or briefly explain why the item is not applicable/relevant for your study

“The aim of the present study is to examine whether an adjunctive, task-shifting component (MIND+) enhances treatment engagement in a mindfulness intervention for stress and depression among Chinese undergraduate and graduate students. Individuals were randomly assigned to a brief (4-week), self-guided, web-based, mindfulness intervention (MIND), or the intervention plus support from non-specialist peer counselors (MIND+). It was hypothesized that at post-treatment, MIND+ participants would show (a) less attrition, (b) greater adherence, (c) greater reductions in stress and depression, and (d) greater increases in mindfulness.”

METHODS

3a) Description of trial design (such as parallel, factorial) including allocation ratio

Does your paper address CONSORT subitem 3a? *

Copy and paste relevant sections from the manuscript (include quotes in quotation marks "like this" to indicate direct quotes from your manuscript), or elaborate on this item by providing additional information not in the ms, or briefly explain why the item is not applicable/relevant for your study

“Participants were 54 currently enrolled university (undergraduate, masters, and doctoral) students from 36 universities across China. Participants randomized to the MIND+ condition (n=27) completed the same procedures as participants in the MIND-only condition (n=27). However, those in the MIND+ condition were informed from the study coordinator via email that they were paired with a peer counselor to provide support and encouragement.”

3b) Important changes to methods after trial commencement (such as eligibility criteria), with reasons

Does your paper address CONSORT subitem 3b? *

Copy and paste relevant sections from the manuscript (include quotes in quotation marks "like this" to indicate direct quotes from your manuscript), or elaborate on this item by providing additional information not in the ms, or briefly explain why the item is not applicable/relevant for your study

Due to delays in some participants receiving the translated materials, day 10 of the trial was adjusted to day 1 to allow all participants to begin the intervention simultaneously.

3b-i) Bug fixes, Downtimes, Content Changes

Bug fixes, Downtimes, Content Changes: ehealth systems are often dynamic systems. A description of changes to methods therefore also includes important changes made on the intervention or comparator during the trial (e.g., major bug fixes or changes in the functionality or content) (5-iii) and other “unexpected events” that may have influenced study design such as staff changes, system failures/downtimes, etc. [2].

	1	2	3	4	5	
subitem not at all important	○	◉	○	○	○	essential

Clear selection

Does your paper address subitem 3b-i?

Copy and paste relevant sections from the manuscript (include quotes in quotation marks "like this" to indicate direct quotes from your manuscript), or elaborate on this item by providing additional information not in the ms, or briefly explain why the item is not applicable/relevant for your study

No notable bugs, delays, or content changes to report aside from what is outlines in item 3b

4a) Eligibility criteria for participants

Does your paper address CONSORT subitem 4a? *

Copy and paste relevant sections from the manuscript (include quotes in quotation marks "like this" to indicate direct quotes from your manuscript), or elaborate on this item by providing additional information not in the ms, or briefly explain why the item is not applicable/relevant for your study

“Inclusion criteria for participants were: (1) Currently enrolled in a university in China (undergraduate, graduate, or doctoral); (2) Has a smartphone and regular access to the internet; (3) Demonstrates the ability to read and understand Mandarin; (4) Reports passing the College English Test—Level 4, at minimum; and (5) Endorses at least mild depression and anxiety. Exclusion criteria were as follows: (1) Under 18-years-old; (2) Does not provide proof of current student status and emergency contact; (3) Endorses current manic or psychotic symptoms; and (4) Endorses suicidal or homicidal ideation during the intake phone interview.

4a-i) Computer / Internet literacy

Computer / Internet literacy is often an implicit “de facto” eligibility criterion - this should be explicitly clarified.

	1	2	3	4	5	
subitem not at all important	○	○	○	○	○	essential

Does your paper address subitem 4a-i?

Copy and paste relevant sections from the manuscript (include quotes in quotation marks "like this" to indicate direct quotes from your manuscript), or elaborate on this item by providing additional information not in the ms, or briefly explain why the item is not applicable/relevant for your study

“Inclusion criteria for participants were as follows:… Has a smartphone and regular access to the internet; Inclusion criteria for peer counselors were as follows:… Has a smartphone and regular access to the internet.”

4a-ii) Open vs. closed, web-based vs. face-to-face assessments:

Open vs. closed, web-based vs. face-to-face assessments: Mention how participants were recruited (online vs. offline), e.g., from an open access website or from a clinic, and clarify if this was a purely web-based trial, or there were face-to-face components (as part of the intervention or for assessment), i.e., to what degree got the study team to know the participant. In online-only trials, clarify if participants were quasi-anonymous and whether having multiple identities was possible or whether technical or logistical measures (e.g., cookies, email confirmation, phone calls) were used to detect/prevent these.

	1	2	3	4	5	
subitem not at all important	○	○	○	◉	○	essential

Clear selection

Does your paper address subitem 4a-ii? *

Copy and paste relevant sections from the manuscript (include quotes in quotation marks "like this" to indicate direct quotes from your manuscript), or elaborate on this item by providing additional information not in the ms, or briefly explain why the item is not applicable/relevant for your study

“Participants were recruited via WeChat blogs, student club listservs, and university websites listing available jobs and research opportunities. Interested individuals completed an online screening assessment. Daily assessments were completed via Qualtrics. Daily questionnaires also assessed participants’ self-reported frequency and duration (in minutes) of mindfulness practice the previous day. A self-report questionnaire packet was completed at screening, baseline, post-intervention, and at 1-month follow-up after the end of the intervention.”

4a-iii) Information giving during recruitment

Information given during recruitment. Specify how participants were briefed for recruitment and in the informed consent procedures (e.g., publish the informed consent documentation as appendix, see also item X26), as this information may have an effect on user self-selection, user expectation and may also bias results.

	1	2	3	4	5	
subitem not at all important	○	○	◉	○	○	essential

Clear selection

Does your paper address subitem 4a-iii?

Copy and paste relevant sections from the manuscript (include quotes in quotation marks "like this" to indicate direct quotes from your manuscript), or elaborate on this item by providing additional information not in the ms, or briefly explain why the item is not applicable/relevant for your study

“Eligible students were contacted by the study coordinator, who conducted a phone interview and orientation to the study procedures. 56 candidates responded to the online survey expressing interest in participating in the study as peer counselors. Those who met inclusion criteria were contacted via telephone to screen for exclusion criteria and to confirm their understanding of the study and willingness to participate in the in-person training and orientation. Volunteer peer counselor candidates who met all criteria for inclusion were invited to the in-person training and orientation. After this training, participants were contacted via telephone to once again assess their willingness to engage in the study.”

4b) Settings and locations where the data were collected

Does your paper address CONSORT subitem 4b? *

Copy and paste relevant sections from the manuscript (include quotes in quotation marks "like this" to indicate direct quotes from your manuscript), or elaborate on this item by providing additional information not in the ms, or briefly explain why the item is not applicable/relevant for your study

“Daily assessments were completed via Qualtrics. A self-report questionnaire packet was completed at screening, baseline, post-intervention, and at 1-month follow-up after the end of the intervention.” The questionnaire packets were completed online and electronically submitted and sent to the research team.

4b-i) Report if outcomes were (self-)assessed through online questionnaires

Clearly report if outcomes were (self-)assessed through online questionnaires (as common in web-based trials) or otherwise.

	1	2	3	4	5	
subitem not at all important	○	○	○	●	○	essential

Clear selection

Does your paper address subitem 4b-i? *

Copy and paste relevant sections from the manuscript (include quotes in quotation marks "like this" to indicate direct quotes from your manuscript), or elaborate on this item by providing additional information not in the ms, or briefly explain why the item is not applicable/relevant for your study

“Daily assessments were completed via Qualtrics. Participants rated their state mindfulness and mood (stress, depression, and happiness) on a 5-point Likert scale (1 = Very Low, 5 = Very High). Daily questionnaires also assessed participants’ self-reported frequency and duration (in minutes) of mindfulness practice the previous day. A self-report questionnaire packet was completed at screening, baseline, post-intervention, and at 1-month follow-up after the end of the intervention.”

4b-ii) Report how institutional affiliations are displayed

Report how institutional affiliations are displayed to potential participants [on ehealth media], as affiliations with prestigious hospitals or universities may affect volunteer rates, use, and reactions with regards to an intervention.(Not a required item – describe only if this may bias results)

	1	2	3	4	5	
subitem not at all important	○	◉	○	○	○	essential

Clear selection

Does your paper address subitem 4b-ii?

Copy and paste relevant sections from the manuscript (include quotes in quotation marks "like this" to indicate direct quotes from your manuscript), or elaborate on this item by providing additional information not in the ms, or briefly explain why the item is not applicable/relevant for your study

The informed consent materials stated that the study was conducted through the Beijing Institute of Technology, and letterhead on Qualtrics informed participants that it involved collaboration with researchers at Duke University.

5) The interventions for each group with sufficient details to allow replication, including how and when they were actually administered

5-i) Mention names, credential, affiliations of the developers, sponsors, and owners

Mention names, credential, affiliations of the developers, sponsors, and owners [6] (if authors/evaluators are owners or developer of the software, this needs to be declared in a "Conflict of interest" section or mentioned elsewhere in the manuscript).

	1	2	3	4	5	
subitem not at all important	○	○	●	○	○	essential

Clear selection

Does your paper address subitem 5-i?

Copy and paste relevant sections from the manuscript (include quotes in quotation marks "like this" to indicate direct quotes from your manuscript), or elaborate on this item by providing additional information not in the ms, or briefly explain why the item is not applicable/relevant for your study

"The "Be Mindful" course is produced by Wellmind Media, with support from the UK-based charity Mental Health Foundation.

5-ii) Describe the history/development process

Describe the history/development process of the application and previous formative evaluations (e.g., focus groups, usability testing), as these will have an impact on adoption/use rates and help with interpreting results.

	1	2	3	4	5	
subitem not at all important	○	○	●	○	○	essential

Clear selection

Does your paper address subitem 5-ii?

Copy and paste relevant sections from the manuscript (include quotes in quotation marks "like this" to indicate direct quotes from your manuscript), or elaborate on this item by providing additional information not in the ms, or briefly explain why the item is not applicable/relevant for your study

“The ‘Be Mindful’ app can be accessed through the website http://www.bemindfulonline.com, where its development and design are fully detailed. To date, 7 peer-reviewed papers have been published reporting study results based on the “Be Mindful” course (for full details, see: https://www.wellmindhealth.com/clinical-studies)... At the time this study was conducted (May 2018), the “Be Mindful” course website reported over 20,000 people had taken the course since 2011.”

5-iii) Revisions and updating

Revisions and updating. Clearly mention the date and/or version number of the application/intervention (and comparator, if applicable) evaluated, or describe whether the intervention underwent major changes during the evaluation process, or whether the development and/or content was “frozen” during the trial. Describe dynamic components such as news feeds or changing content which may have an impact on the replicability of the intervention (for unexpected events see item 3b).

	1	2	3	4	5	
subitem not at all important	○	◉	○	○	○	essential

Clear selection

Does your paper address subitem 5-iii?

Copy and paste relevant sections from the manuscript (include quotes in quotation marks "like this" to indicate direct quotes from your manuscript), or elaborate on this item by providing additional information not in the ms, or briefly explain why the item is not applicable/relevant for your study

The researchers used the most up-to-date version of the “Be Mindful” intervention during the May 2018 study. There were no major changes in the software during the course of the study.

5-iv) Quality assurance methods

Provide information on quality assurance methods to ensure accuracy and quality of information provided [1], if applicable.

	1	2	3	4	5	
subitem not at all important	○	○	●	○	○	essential

Clear selection

Does your paper address subitem 5-iv?

Copy and paste relevant sections from the manuscript (include quotes in quotation marks "like this" to indicate direct quotes from your manuscript), or elaborate on this item by providing additional information not in the ms, or briefly explain why the item is not applicable/relevant for your study

“Project staff received technical support and administrative access to randomize participants, track progress (eg, participant logins, module completion, date of completion), and download data. All materials on the course were translated into Mandarin, including the videos, audio recordings, and homework assignments. Each week, the research coordinators emailed materials to participants after they were ready to progress to the next chapter in the course.”

5-v) Ensure replicability by publishing the source code, and/or providing screenshots/screen-capture video, and/or providing flowcharts of the algorithms used

Ensure replicability by publishing the source code, and/or providing screenshots/screen-capture video, and/or providing flowcharts of the algorithms used. Replicability (i.e., other researchers should in principle be able to replicate the study) is a hallmark of scientific reporting.

	1	2	3	4	5	
subitem not at all important	○	○	●	○	○	essential

Clear selection

Does your paper address subitem 5-v?

Copy and paste relevant sections from the manuscript (include quotes in quotation marks "like this" to indicate direct quotes from your manuscript), or elaborate on this item by providing additional information not in the ms, or briefly explain why the item is not applicable/relevant for your study

N/A

5-vi) Digital preservation

Digital preservation: Provide the URL of the application, but as the intervention is likely to change or disappear over the course of the years; also make sure the intervention is archived (Internet Archive, webcitation.org, and/or publishing the source code or screenshots/videos alongside the article). As pages behind login screens cannot be archived, consider creating demo pages which are accessible without login.

	1	2	3	4	5	
subitem not at all important	○	○	◉	○	○	essential

Clear selection

Does your paper address subitem 5-vi?

Copy and paste relevant sections from the manuscript (include quotes in quotation marks "like this" to indicate direct quotes from your manuscript), or elaborate on this item by providing additional information not in the ms, or briefly explain why the item is not applicable/relevant for your study

"The 'Be Mindful' program can be accessed through the website http://www.bemindfulonline.com." The archived link is: https://webcitation.org/6Ot8VkvVB.

5-vii) Access

Access: Describe how participants accessed the application, in what setting/context, if they had to pay (or were paid) or not, whether they had to be a member of specific group. If known, describe how participants obtained “access to the platform and Internet” [1]. To ensure access for editors/reviewers/readers, consider to provide a “backdoor” login account or demo mode for reviewers/readers to explore the application (also important for archiving purposes, see vi).

	1	2	3	4	5	
subitem not at all important	○	○	○	◉	○	essential

Clear selection

Does your paper address subitem 5-vii? *

Copy and paste relevant sections from the manuscript (include quotes in quotation marks "like this" to indicate direct quotes from your manuscript), or elaborate on this item by providing additional information not in the ms, or briefly explain why the item is not applicable/relevant for your study

“Participants in the current study were able to complete the course for free. Project staff received technical support and administrative access to randomize participants, track progress (eg, participant logins, module completion, date of completion), and download data. All materials on the course were translated into Mandarin, including the videos, audio recordings, and homework assignments. Each week, the research coordinators emailed materials to participants after they were ready to progress to the next chapter in the course.”

5-viii) Mode of delivery, features/functionalities/components of the intervention and comparator, and the theoretical framework

Describe mode of delivery, features/functionalities/components of the intervention and comparator, and the theoretical framework [6] used to design them (instructional strategy [1], behaviour change techniques, persuasive features, etc., see e.g., [7, 8] for terminology). This includes an in-depth description of the content (including where it is coming from and who developed it) [1],” whether [and how] it is tailored to individual circumstances and allows users to track their progress and receive feedback” [6]. This also includes a description of communication delivery channels and – if computer-mediated communication is a component – whether communication was synchronous or asynchronous [6]. It also includes information on presentation strategies [1], including page design principles, average amount of text on pages, presence of hyperlinks to other resources, etc. [1].

	1	2	3	4	5	
subitem not at all important	○	○	○	◉	○	essential

Clear selection

Does your paper address subitem 5-viii? *

Copy and paste relevant sections from the manuscript (include quotes in quotation marks "like this" to indicate direct quotes from your manuscript), or elaborate on this item by providing additional information not in the ms, or briefly explain why the item is not applicable/relevant for your study

“’Be Mindful’ delivers all the elements of MBCT in an internet-based course that can be completed in 4 weeks. An overview of course content is listed in Table 1. Project staff received technical support and administrative access to randomize participants, track progress, and download data. All materials on the course were translated into Mandarin, including the videos, audio recordings, and homework assignments. Each week, the research coordinators emailed materials to participants after they were ready to progress to the next chapter in the course. MIND+ participants completed the same procedures as participants in the MIND-only condition. However, those in the MIND+ condition were informed from the study coordinator via email that they were paired with a peer counselor to provide support and encouragement.

5-ix) Describe use parameters

Describe use parameters (e.g., intended “doses” and optimal timing for use). Clarify what instructions or recommendations were given to the user, e.g., regarding timing, frequency, heaviness of use, if any, or was the intervention used ad libitum.

	1	2	3	4	5	
subitem not at all important	○	○	○	◉	○	essential

Clear selection

Does your paper address subitem 5-ix?

Copy and paste relevant sections from the manuscript (include quotes in quotation marks "like this" to indicate direct quotes from your manuscript), or elaborate on this item by providing additional information not in the ms, or briefly explain why the item is not applicable/relevant for your study

“‘Be Mindful’ delivers all the elements of MBCT in an internet-based course that can be completed in 4 weeks. Daily assessments were completed via Qualtrics. Participants rated their state mindfulness and mood (stress, depression, and happiness) on a 5-point Likert scale (1 = Very Low, 5 = Very High). Daily questionnaires also assessed participants’ self-reported frequency and duration (in minutes) of mindfulness practice the previous day. Links to questionnaires expired within 4 hours if not completed.”

5-x) Clarify the level of human involvement

Clarify the level of human involvement (care providers or health professionals, also technical assistance) in the e-intervention or as co-intervention (detail number and expertise of professionals involved, if any, as well as “type of assistance offered, the timing and frequency of the support, how it is initiated, and the medium by which the assistance is delivered”. It may be necessary to distinguish between the level of human involvement required for the trial, and the level of human involvement required for a routine application outside of a RCT setting (discuss under item 21 – generalizability).

	1	2	3	4	5	
subitem not at all important	○	○	◉	○	○	essential

Clear selection

Does your paper address subitem 5-x?

Copy and paste relevant sections from the manuscript (include quotes in quotation marks "like this" to indicate direct quotes from your manuscript), or elaborate on this item by providing additional information not in the ms, or briefly explain why the item is not applicable/relevant for your study

"The 'Be Mindful' course is self-guided; in other words, there is no contact with mindfulness teachers or other course participants. Each week, the research coordinators emailed materials to participants after they were ready to progress to the next chapter in the course. Peer counselors were encouraged to provide brief (15-20 minute) weekly meetings to support and encourage participants in their completion of the internet-based intervention."

5-xi) Report any prompts/reminders used

Report any prompts/reminders used: Clarify if there were prompts (letters, emails, phone calls, SMS) to use the application, what triggered them, frequency etc. It may be necessary to distinguish between the level of prompts/reminders required for the trial, and the level of prompts/reminders for a routine application outside of a RCT setting (discuss under item 21 – generalizability).

	1	2	3	4	5	
subitem not at all important	○	○	◉	○	○	essential

Clear selection

Does your paper address subitem 5-xi? *

Copy and paste relevant sections from the manuscript (include quotes in quotation marks "like this" to indicate direct quotes from your manuscript), or elaborate on this item by providing additional information not in the ms, or briefly explain why the item is not applicable/relevant for your study

"Those in the MIND+ condition were informed from the study coordinator via email that they were paired with a peer counselor. Peer counselors were instructed to contact their participants if they did not hear from them within 5 days. Peer counselors were encouraged to provide brief weekly meetings to support and encourage participants in their completion of the intervention. 6 weeks after initial enrollment, participants received an email thanking them for their participation and a link inviting them to complete the post-treatment assessment. If participants did not complete the survey within 1 week they were contacted over the course of the next week." The "Be Mindful Online" app also sent participants weekly prompts to encourage them to begin work on the upcoming module.

5-xii) Describe any co-interventions (incl. training/support)

Describe any co-interventions (incl. training/support): Clearly state any interventions that are provided in addition to the targeted eHealth intervention, as ehealth intervention may not be designed as stand-alone intervention. This includes training sessions and support [1]. It may be necessary to distinguish between the level of training required for the trial, and the level of training for a routine application outside of a RCT setting (discuss under item 21 – generalizability.

	1	2	3	4	5	
subitem not at all important	○	○	◉	○	○	essential

Clear selection

Does your paper address subitem 5-xii? *

Copy and paste relevant sections from the manuscript (include quotes in quotation marks "like this" to indicate direct quotes from your manuscript), or elaborate on this item by providing additional information not in the ms, or briefly explain why the item is not applicable/relevant for your study

“In-person peer counselor training took place for 8 hours. Candidates were given opportunities to practice using skills in dyads, and to receive coaching and feedback from the first author (M.R.) and research assistants. Weekly group supervision was attended by the M.R., 2 research assistants, and the 4 peer counselors. The meetings began with a brief mindfulness practice and discussion of observations. Team members presented consultation questions and supported each other using peer counseling techniques, in an effort to enhance capabilities and motivation. M.R. provided 5- to 10-minute didactic lessons related to common challenges peer counselors were facing. Peer counselors were offered the opportunity to schedule additional individual supervision from the study coordinator on an as-needed basis, or in case of a participant emergency."

6a) Completely defined pre-specified primary and secondary outcome measures, including how and when they were assessed

Does your paper address CONSORT subitem 6a? *

Copy and paste relevant sections from the manuscript (include quotes in quotation marks "like this" to indicate direct quotes from your manuscript), or elaborate on this item by providing additional information not in the ms, or briefly explain why the item is not applicable/relevant for your study

“Attrition was indicated by higher completion rates of the post-treatment assessment. Adherence was indicated by more frequent use of the course (total log-ins), and a larger % of the course completed. Engagement levels were indicated by (1) likelihood of responding to daily prompts & (2) likelihood of reporting daily practice. A self-report survey was completed at screening, baseline, post-intervention, and a 1-month follow-up” and included: “Demographic Data Survey – Modified (DDS-M); Generalized Anxiety Disorder Questionnaire (GAD-7); Patient Health Questionnaire (PHQ-9); Five-Factor Mindfulness Questionnaire (FFMQ); Depression Anxiety Stress Scale (DASS-21); Perceived Stress Scale (PSS); and a "Perceived Barriers to Mindfulness Practice" self-report measure. Items are presented in Appendix A.” Data collection was complete in May 2018, and all data was analyzed within 1 month.

6a-i) Online questionnaires: describe if they were validated for online use and apply CHERRIES items to describe how the questionnaires were designed/deployed

If outcomes were obtained through online questionnaires, describe if they were validated for online use and apply CHERRIES items to describe how the questionnaires were designed/deployed [9].

	1	2	3	4	5	
subitem not at all important	○	○	◉	○	○	essential

Clear selection

Does your paper address subitem 6a-i?

Copy and paste relevant sections from manuscript text

While the questionnaires were not specifically validated for online use, they included open-source, popular measures (see item 6a).

6a-ii) Describe whether and how “use” (including intensity of use/dosage) was defined/measured/monitored

Describe whether and how “use” (including intensity of use/dosage) was defined/measured/monitored (logins, logfile analysis, etc.). Use/adoption metrics are important process outcomes that should be reported in any ehealth trial.

	1	2	3	4	5	
subitem not at all important	○	○	○	◉	○	essential

Clear selection

Does your paper address subitem 6a-ii?

Copy and paste relevant sections from manuscript text

Usage was measured through “daily questionnaires which assessed participants’ self-reported frequency and duration (in minutes) of mindfulness practice the previous day.” Additionally, “project staff received administrative access to track progress (eg, participant logins, module completion, date of completion), and download data.

6a-iii) Describe whether, how, and when qualitative feedback from participants was obtained

Describe whether, how, and when qualitative feedback from participants was obtained (e.g., through emails, feedback forms, interviews, focus groups).

	1	2	3	4	5	
subitem not at all important	○	○	◉	○	○	essential

Clear selection

Does your paper address subitem 6a-iii?

Copy and paste relevant sections from manuscript text

In the post-treatment questionnaires, both peer counselors and participants had the opportunity to provide qualitative acceptability and feasibility feedback.

6b) Any changes to trial outcomes after the trial commenced, with reasons

Does your paper address CONSORT subitem 6b? *

Copy and paste relevant sections from the manuscript (include quotes in quotation marks "like this" to indicate direct quotes from your manuscript), or elaborate on this item by providing additional information not in the ms, or briefly explain why the item is not applicable/relevant for your study

There were no changes to trial outcomes following commencement of the trial.

7a) How sample size was determined

NPT: When applicable, details of whether and how the clustering by care provides or centers was addressed

7a-i) Describe whether and how expected attrition was taken into account when calculating the sample size

Describe whether and how expected attrition was taken into account when calculating the sample size.

	1	2	3	4	5	
subitem not at all important	○	○	◉	○	○	essential

Clear selection

Does your paper address subitem 7a-i?

Copy and paste relevant sections from manuscript title (include quotes in quotation marks "like this" to indicate direct quotes from your manuscript), or elaborate on this item by providing additional information not in the ms, or briefly explain why the item is not applicable/relevant for your study

Attrition was not accounted for when calculating sample size. There was no pre-established sample size; it was simply the total number of interested candidates who met all inclusion criteria.

7b) When applicable, explanation of any interim analyses and stopping guidelines

Does your paper address CONSORT subitem 7b? *

Copy and paste relevant sections from the manuscript (include quotes in quotation marks "like this" to indicate direct quotes from your manuscript), or elaborate on this item by providing additional information not in the ms, or briefly explain why the item is not applicable/relevant for your study

There were no interim analyses or stopping guidelines in this study.

8a) Method used to generate the random allocation sequence

NPT: When applicable, how care providers were allocated to each trial group

Does your paper address CONSORT subitem 8a? *

Copy and paste relevant sections from the manuscript (include quotes in quotation marks "like this" to indicate direct quotes from your manuscript), or elaborate on this item by providing additional information not in the ms, or briefly explain why the item is not applicable/relevant for your study

“Project staff received technical support and administrative access to randomize participants” which was accomplished via a digital randomization program.

8b) Type of randomisation; details of any restriction (such as blocking and block size)

Does your paper address CONSORT subitem 8b? *

Copy and paste relevant sections from the manuscript (include quotes in quotation marks "like this" to indicate direct quotes from your manuscript), or elaborate on this item by providing additional information not in the ms, or briefly explain why the item is not applicable/relevant for your study

Using random.org, two 25-person blocks were used to randomize the participants into 2 equally-sized groups.

9) Mechanism used to implement the random allocation sequence (such as sequentially numbered containers), describing any steps taken to conceal the sequence until interventions were assigned

Does your paper address CONSORT subitem 9? *

Copy and paste relevant sections from the manuscript (include quotes in quotation marks "like this" to indicate direct quotes from your manuscript), or elaborate on this item by providing additional information not in the ms, or briefly explain why the item is not applicable/relevant for your study

The automated, web-based randomization service (random.org) used in this study generates randomness via atmospheric noise.

10) Who generated the random allocation sequence, who enrolled participants, and who assigned participants to interventions

Does your paper address CONSORT subitem 10? *

Copy and paste relevant sections from the manuscript (include quotes in quotation marks "like this" to indicate direct quotes from your manuscript), or elaborate on this item by providing additional information not in the ms, or briefly explain why the item is not applicable/relevant for your study

First author M.R. and project staff coordinated with “interested individuals who completed an online screening assessment. Eligible students were contacted by M.R., who conducted a phone interview and orientation to the study procedures. Students who provided proof of student status and emergency contact information received a link to the baseline assessment measures. Students who completed the online baseline assessment measures were randomly assigned to a brief, 4-week internet-based mindfulness intervention (MIND), or to the intervention plus peer counselor support (MIND+).” The randomization sequence was sourced through random.org, an automated, web-based randomization service which generates randomness via atmospheric noise.

11a) If done, who was blinded after assignment to interventions (for example, participants, care providers, those assessing outcomes) and how

NPT: Whether or not administering co-interventions were blinded to group assignment

11a-i) Specify who was blinded, and who wasn’t

Specify who was blinded, and who wasn’t. Usually, in web-based trials it is not possible to blind the participants [1, 3] (this should be clearly acknowledged), but it may be possible to blind outcome assessors, those doing data analysis or those administering co-interventions (if any).

	1	2	3	4	5	
subitem not at all important	○	○	○	◉	○	essential

Clear selection

Does your paper address subitem 11a-i? *

Copy and paste relevant sections from the manuscript (include quotes in quotation marks "like this" to indicate direct quotes from your manuscript), or elaborate on this item by providing additional information not in the ms, or briefly explain why the item is not applicable/relevant for your study

There was no blinding of participants, peer counselors, outcome assessors, or data analysts in this study.

11a-ii) Discuss e.g., whether participants knew which intervention was the “intervention of interest” and which one was the “comparator”

Informed consent procedures (4a-ii) can create biases and certain expectations - discuss e.g., whether participants knew which intervention was the “intervention of interest” and which one was the “comparator”.

	1	2	3	4	5	
subitem not at all important	○	○	○	◉	○	essential

Clear selection

Does your paper address subitem 11a-ii?

Copy and paste relevant sections from the manuscript (include quotes in quotation marks "like this" to indicate direct quotes from your manuscript), or elaborate on this item by providing additional information not in the ms, or briefly explain why the item is not applicable/relevant for your study

No, participants were unaware of the full study design and each group received a separate informed consent form tailored to their specific intervention.

11b) If relevant, description of the similarity of interventions

(this item is usually not relevant for ehealth trials as it refers to similarity of a placebo or sham intervention to a active medication/intervention)

Does your paper address CONSORT subitem 11b? *

Copy and paste relevant sections from the manuscript (include quotes in quotation marks "like this" to indicate direct quotes from your manuscript), or elaborate on this item by providing additional information not in the ms, or briefly explain why the item is not applicable/relevant for your study

Both groups received the same internet-based mindfulness intervention, the only difference was that one group received the intervention plus peer counselor support (MIND+), while the other received no peer counselor support (MIND).

12a) Statistical methods used to compare groups for primary and secondary outcomes

NPT: When applicable, details of whether and how the clustering by care providers or centers was addressed

Does your paper address CONSORT subitem 12a? *

Copy and paste relevant sections from the manuscript (include quotes in quotation marks "like this" to indicate direct quotes from your manuscript), or elaborate on this item by providing additional information not in the ms, or briefly explain why the item is not applicable/relevant for your study

“A $\chi 2$ analysis assessed if MIND+ participants showed less attrition. Independent samples t-tests assessed if MIND+ participants showed greater adherence. Logistic multilevel models assessed if MIND+ participants showed a less robust decrease over time in (1) Responses to daily prompts & (2) likelihood of reporting daily practice. Both outcomes were predicted from condition, time, & interaction. Time was alternatively defined by contrasting the beginning of the study with the middle and end. A multilevel growth model assessed if MIND+ participants showed a less robust decrease over time in the number of minutes of daily practice. The model predicted daily number of minutes from condition, time, & interaction. 3 multilevel models assessed if MIND+ participants showed a greater increase in mindfulness, and greater decreases in depression and stress."

12a-i) Imputation techniques to deal with attrition / missing values

Imputation techniques to deal with attrition / missing values: Not all participants will use the intervention/comparator as intended and attrition is typically high in ehealth trials. Specify how participants who did not use the application or dropped out from the trial were treated in the statistical analysis (a complete case analysis is strongly discouraged, and simple imputation techniques such as LOCF may also be problematic [4]).

	1	2	3	4	5	
subitem not at all important	○	○	○	◉	○	essential

Clear selection

Does your paper address subitem 12a-i? *

Copy and paste relevant sections from the manuscript (include quotes in quotation marks "like this" to indicate direct quotes from your manuscript), or elaborate on this item by providing additional information not in the ms, or briefly explain why the item is not applicable/relevant for your study

Attrition was one of the outcomes measured and analyzed, and was “indicated by higher completion (vs. non-completion) rates of post-treatment assessment.” Also, Maximum likelihood estimation (MLE) and multiple imputation (MI) were used.

12b) Methods for additional analyses, such as subgroup analyses and adjusted analyses

Does your paper address CONSORT subitem 12b? *

Copy and paste relevant sections from the manuscript (include quotes in quotation marks "like this" to indicate direct quotes from your manuscript), or elaborate on this item by providing additional information not in the ms, or briefly explain why the item is not applicable/relevant for your study

“Person-standardized daily values (today’s value minus overall person mean, divided by overall person standard deviation) were utilized for graphical depictions of continuous outcomes in order to depict only the within-person changes in the outcome across the study, consistent with multilevel modeling results.”

X26) REB/IRB Approval and Ethical Considerations [recommended as subheading under "Methods"] (not a CONSORT item)

X26-i) Comment on ethics committee approval

	1	2	3	4	5	
subitem not at all important	○	○	○	○	◉	essential

Clear selection

Does your paper address subitem X26-i?

Copy and paste relevant sections from the manuscript (include quotes in quotation marks "like this" to indicate direct quotes from your manuscript), or elaborate on this item by providing additional information not in the ms, or briefly explain why the item is not applicable/relevant for your study

“This study received IRB approval from the Psychology Research Ethics Committee at the Beijing Institute of Technology.”

x26-ii) Outline informed consent procedures

Outline informed consent procedures e.g., if consent was obtained offline or online (how? Checkbox, etc.?), and what information was provided (see 4a-ii). See [6] for some items to be included in informed consent documents.

	1	2	3	4	5	
subitem not at all important	○	○	○	○	●	essential

Clear selection

Does your paper address subitem X26-ii?

Copy and paste relevant sections from the manuscript (include quotes in quotation marks "like this" to indicate direct quotes from your manuscript), or elaborate on this item by providing additional information not in the ms, or briefly explain why the item is not applicable/relevant for your study

“All participants electronically signed a digital informed consent form," which will be published as an appendix.

X26-iii) Safety and security procedures

Safety and security procedures, incl. privacy considerations, and any steps taken to reduce the likelihood or detection of harm (e.g., education and training, availability of a hotline)

	1	2	3	4	5	
subitem not at all important	○	○	○	●	○	essential

Clear selection

Does your paper address subitem X26-iii?

Copy and paste relevant sections from the manuscript (include quotes in quotation marks "like this" to indicate direct quotes from your manuscript), or elaborate on this item by providing additional information not in the ms, or briefly explain why the item is not applicable/relevant for your study

Peer counselors were trained to address life-threatening behaviors, and the initial screening assessed suicidal ideation. The only cybersecurity measures in place were those provided by “Be Mindful Online” and Qualtrics.

RESULTS

13a) For each group, the numbers of participants who were randomly assigned, received intended treatment, and were analysed for the primary outcome

NPT: The number of care providers or centers performing the intervention in each group and the number of patients treated by each care provider in each center

Does your paper address CONSORT subitem 13a? *

Copy and paste relevant sections from the manuscript (include quotes in quotation marks "like this" to indicate direct quotes from your manuscript), or elaborate on this item by providing additional information not in the ms, or briefly explain why the item is not applicable/relevant for your study

“Figure 1 is a flow chart illustrating participant flow from recruitment to study completion. Table 2 provides descriptive statistics for demographics and key study variables, for both the total sample and for each condition.” 27 participants were assigned to each condition and all 54 initially received their intended treatment. 7 MIND participants completed the intervention, whereas 16 MIND+ participants completed the intervention. Data from all 54 participants were included in primary outcome analyses. 4 individuals served as peer counselors for participants in the MIND+ group. The principal investigator (M.R.) and 2 research assistants facilitated the study.

13b) For each group, losses and exclusions after randomisation, together with reasons

Does your paper address CONSORT subitem 13b? (NOTE: Preferably, this is shown in a CONSORT flow diagram) *

Copy and paste relevant sections from the manuscript (include quotes in quotation marks "like this" to indicate direct quotes from your manuscript), or elaborate on this item by providing additional information not in the ms, or briefly explain why the item is not applicable/relevant for your study

No participants were lost or excluded following randomization, aside from those who dropped out at various stages on their own volition. 27 participants were initially randomized to each condition, with 7 in the MIND group completing the entire intervention and 16 in the MIND+ group completing the entire intervention. Thus, 23 out of the 54 initial participants completed the entire intervention, with no explanation as to why the other 31 dropped out at various stages during the intervention. A schematic is available in ms Fig. 1.

13b-i) Attrition diagram

Strongly recommended: An attrition diagram (e.g., proportion of participants still logging in or using the intervention/comparator in each group plotted over time, similar to a survival curve) or other figures or tables demonstrating usage/dose/engagement.

	1	2	3	4	5	
subitem not at all important	○	○	◉	○	○	essential

Clear selection

Does your paper address subitem 13b-i?

Copy and paste relevant sections from the manuscript or cite the figure number if applicable (include quotes in quotation marks "like this" to indicate direct quotes from your manuscript), or elaborate on this item by providing additional information not in the ms, or briefly explain why the item is not applicable/relevant for your study

N/A

14a) Dates defining the periods of recruitment and follow-up

Does your paper address CONSORT subitem 14a? *

Copy and paste relevant sections from the manuscript (include quotes in quotation marks "like this" to indicate direct quotes from your manuscript), or elaborate on this item by providing additional information not in the ms, or briefly explain why the item is not applicable/relevant for your study

Recruitment commenced on August 5th, 2017, and data collection was completed on April 7th, 2018.

14a-i) Indicate if critical “secular events” fell into the study period

Indicate if critical “secular events” fell into the study period, e.g., significant changes in Internet resources available or “changes in computer hardware or Internet delivery resources”

	1	2	3	4	5	
subitem not at all important	◉	○	○	○	○	essential

Clear selection

Does your paper address subitem 14a-i?

Copy and paste relevant sections from the manuscript (include quotes in quotation marks "like this" to indicate direct quotes from your manuscript), or elaborate on this item by providing additional information not in the ms, or briefly explain why the item is not applicable/relevant for your study

No noteworthy “secular events” affected or interfered with the study period.

14b) Why the trial ended or was stopped (early)

Does your paper address CONSORT subitem 14b? *

Copy and paste relevant sections from the manuscript (include quotes in quotation marks "like this" to indicate direct quotes from your manuscript), or elaborate on this item by providing additional information not in the ms, or briefly explain why the item is not applicable/relevant for your study

The trial progressed according to plan without interruption, and was considered finished “Six weeks after initial enrollment,” as originally scheduled. Additionally, participants were asked to fill out a final “self-report questionnaire packet… at [a] 1-month follow-up after the end of the intervention.”

15) A table showing baseline demographic and clinical characteristics for each group

NPT: When applicable, a description of care providers (case volume, qualification, expertise, etc.) and centers (volume) in each group

Does your paper address CONSORT subitem 15? *

Copy and paste relevant sections from the manuscript (include quotes in quotation marks "like this" to indicate direct quotes from your manuscript), or elaborate on this item by providing additional information not in the ms, or briefly explain why the item is not applicable/relevant for your study

See Table 2 in MS

15-i) Report demographics associated with digital divide issues

In ehealth trials it is particularly important to report demographics associated with digital divide issues, such as age, education, gender, social-economic status, computer/Internet/ehealth literacy of the participants, if known.

	1	2	3	4	5	
subitem not at all important	○	○	●	○	○	essential

Clear selection

Does your paper address subitem 15-i? *

Copy and paste relevant sections from the manuscript (include quotes in quotation marks "like this" to indicate direct quotes from your manuscript), or elaborate on this item by providing additional information not in the ms, or briefly explain why the item is not applicable/relevant for your study

Demographic info on age and gender and provided in the response to item 15 above. All participants were Chinese university students enrolled in either undergraduate (n=21), master’s (n=29), or doctoral (n=4) programs. All participants acknowledged that they passed “the College English Test—Level 4, at minimum” and had “a smartphone and regular access to the internet.”

16) For each group, number of participants (denominator) included in each analysis and whether the analysis was by original assigned groups

16-i) Report multiple “denominators” and provide definitions

Report multiple “denominators” and provide definitions: Report N’s (and effect sizes) “across a range of study participation [and use] thresholds” [1], e.g., N exposed, N consented, N used more than x times, N used more than y weeks, N participants “used” the intervention/comparator at specific pre-defined time points of interest (in absolute and relative numbers per group). Always clearly define “use” of the intervention.

	1	2	3	4	5	
subitem not at all important	○	○	○	◉	○	essential

Clear selection

Does your paper address subitem 16-i? *

Copy and paste relevant sections from the manuscript (include quotes in quotation marks "like this" to indicate direct quotes from your manuscript), or elaborate on this item by providing additional information not in the ms, or briefly explain why the item is not applicable/relevant for your study

n=27 participants were randomized to both the MIND and MIND+ conditions, for an overall sample size of N=54. Overall, n=23 participants completed the intervention, with n=7 from the MIND group and n=16 from the MIND+ group.

16-ii) Primary analysis should be intent-to-treat

Primary analysis should be intent-to-treat, secondary analyses could include comparing only “users”, with the appropriate caveats that this is no longer a randomized sample (see 18-i).

	1	2	3	4	5	
subitem not at all important	○	○	○	◉	○	essential

Clear selection

Does your paper address subitem 16-ii?

Copy and paste relevant sections from the manuscript (include quotes in quotation marks "like this" to indicate direct quotes from your manuscript), or elaborate on this item by providing additional information not in the ms, or briefly explain why the item is not applicable/relevant for your study

All primary analyses in this study are intent-to-treat, meaning that each analysis included the n=27 participants randomized to each condition (MIND and MIND+).

17a) For each primary and secondary outcome, results for each group, and the estimated effect size and its precision (such as 95% confidence interval)

Does your paper address CONSORT subitem 17a? *

Copy and paste relevant sections from the manuscript (include quotes in quotation marks "like this" to indicate direct quotes from your manuscript), or elaborate on this item by providing additional information not in the ms, or briefly explain why the item is not applicable/relevant for your study

A χ2 analysis revealed more completers in MIND+ (χ21=6.13, P=.013; hypothesis 1). An independent samples t-test yielded a higher mean percentage of course completion in MIND+ (Mean Difference 21.85, 95% CI for MD 42.69 to 1.010; t52=2.10, P=.041; hypothesis 2) Multilevel models reveal that both stress (t39.4=-3.90, P<.01, SE 0.006) and depression (t39.2=-2.40, P<.05, SE 0.005) decreased linearly as study day increased, but the linear effect did not differ by condition (t35.2=-1.04, SE 0.268; t39.1=-0.59 SE 0.273; hypothesis 3). There were no linear effects of study day on daily mindfulness (t41.2=0.30, SE 0.005), and the effect did not differ by condition (t42=0.03, SE 0.237). For daily outcomes of stress and depression, MIND+ participants showed a significantly greater decline from study phase 1 to 3 (t266=-2.07, P<.05, SE 0.113; t265=-2.19, P<.05, SE 0.128).

17a-i) Presentation of process outcomes such as metrics of use and intensity of use

In addition to primary/secondary (clinical) outcomes, the presentation of process outcomes such as metrics of use and intensity of use (dose, exposure) and their operational definitions is critical. This does not only refer to metrics of attrition (13-b) (often a binary variable), but also to more continuous exposure metrics such as “average session length”. These must be accompanied by a technical description how a metric like a “session” is defined (e.g., timeout after idle time) [1] (report under item 6a).

	1	2	3	4	5	
subitem not at all important	○	○	○	●	○	essential

Clear selection

Does your paper address subitem 17a-i?

Copy and paste relevant sections from the manuscript (include quotes in quotation marks "like this" to indicate direct quotes from your manuscript), or elaborate on this item by providing additional information not in the ms, or briefly explain why the item is not applicable/relevant for your study

Percentage of course completed: Full sample (N=54, mean 61.66, SD 39.37); MIND (n=27, mean 50.74, SD 37.40); MIND+ (n=27, mean 72.59, SD 38.88); comparison (t52=-2.10, P=.040). Number of logins: Full sample (N=54, mean 15.72, SD 12.35); MIND (n=27, mean 13.92, SD 9.30); MIND+ (n=27, mean 17.51, SD 12.39); comparison (t52 = -1.07, P=.289). “MIND+ participants… demonstrated a non-significant trend toward lower rates of non-use attrition (P=.051), defined as never responding to the daily assessment and/or not responding for at least the last 3 weeks of the study.”

17b) For binary outcomes, presentation of both absolute and relative effect sizes is recommended

Does your paper address CONSORT subitem 17b? *

Copy and paste relevant sections from the manuscript (include quotes in quotation marks "like this" to indicate direct quotes from your manuscript), or elaborate on this item by providing additional information not in the ms, or briefly explain why the item is not applicable/relevant for your study

Yes, attrition, i.e., completion of both pre and post-treatment assessment measures. "). "A chi-squared analysis comparing dichotomous condition assignment (MIND vs. MIND+) and post-treatment assessment (completed vs. not completed) revealed a greater number of completers in the MIND+ condition ($\chi 21=6.13$, P=.013)."

18) Results of any other analyses performed, including subgroup analyses and adjusted analyses, distinguishing pre-specified from exploratory

Does your paper address CONSORT subitem 18? *

Copy and paste relevant sections from the manuscript (include quotes in quotation marks "like this" to indicate direct quotes from your manuscript), or elaborate on this item by providing additional information not in the ms, or briefly explain why the item is not applicable/relevant for your study

“Participants in the present study presented with mean baseline PSS (stress), GAD-7 (anxiety), and PHQ-9 (depression) scores of 23.27 (SD 4.28), 9.90 (SD 3.98), and 11.31 (SD 5.06), respectively… The pre-post effect sizes (d) for stress among completers (from both MIND and MIND+) was 1.13… The pre-post effect sizes (d) for anxiety (GAD-7) and depression (PHQ-9) among completers (from both MIND and MIND+) were 0.89 and 0.95, respectively… MIND+ participants did not report significant improvements in daily ratings of state mindfulness across the trial compared to participants in the MIND-only condition. Instead, there was a main effect of treatment on improvements on daily ratings of mindfulness. In the same way, there were not between-group differences in pre-post FFMQ scores— though there was a moderate main effect for mindfulness (FFMQ) among completers (d=0.55).”

18-i) Subgroup analysis of comparing only users

A subgroup analysis of comparing only users is not uncommon in ehealth trials, but if done, it must be stressed that this is a self-selected sample and no longer an unbiased sample from a randomized trial (see 16-iii).

	1	2	3	4	5	
subitem not at all important	○	○	○	◉	○	essential

Clear selection

Does your paper address subitem 18-i?

Copy and paste relevant sections from the manuscript (include quotes in quotation marks "like this" to indicate direct quotes from your manuscript), or elaborate on this item by providing additional information not in the ms, or briefly explain why the item is not applicable/relevant for your study

"Each participant in the MIND+ group… received a mean of 4.69 peer counseling "sessions" (SD 2.04; range 0-7), lasting an average total of 120.85 minutes (SD 66.53; range 12-345 minutes) per MIND+ participant.

19) All important harms or unintended effects in each group

(for specific guidance see CONSORT for harms)

Does your paper address CONSORT subitem 19? *

Copy and paste relevant sections from the manuscript (include quotes in quotation marks "like this" to indicate direct quotes from your manuscript), or elaborate on this item by providing additional information not in the ms, or briefly explain why the item is not applicable/relevant for your study

"9% (4/43) of participants indicated that language was 'very much' a barrier to completion. It is also possible that language was more of a barrier for the 11 participants who did not complete the post-treatment assessment. However, follow-up analyses did not reveal an association between language ability and course completion or engagement. Specifically, follow-up analyses revealed that self-reported baseline English proficiency and post-treatment perception of language as a barrier to completion were not significant predictors of treatment completion."

19-i) Include privacy breaches, technical problems

Include privacy breaches, technical problems. This does not only include physical “harm” to participants, but also incidents such as perceived or real privacy breaches [1], technical problems, and other unexpected/unintended incidents. “Unintended effects” also includes unintended positive effects [2].

	1	2	3	4	5	
subitem not at all important	○	○	◉	○	○	essential

Clear selection

Does your paper address subitem 19-i?

Copy and paste relevant sections from the manuscript (include quotes in quotation marks "like this" to indicate direct quotes from your manuscript), or elaborate on this item by providing additional information not in the ms, or briefly explain why the item is not applicable/relevant for your study

There are no privacy breaches to report. The response to item 3b covers the only notable technical problem, which involves delays in some participants receiving study materials, which led to a 10-day delay in the study’s schedule.

19-ii) Include qualitative feedback from participants or observations from staff/researchers

Include qualitative feedback from participants or observations from staff/researchers, if available, on strengths and shortcomings of the application, especially if they point to unintended/unexpected effects or uses. This includes (if available) reasons for why people did or did not use the application as intended by the developers.

	1	2	3	4	5	
subitem not at all important	○	○	◉	○	○	essential

Clear selection

Does your paper address subitem 19-ii?

Copy and paste relevant sections from the manuscript (include quotes in quotation marks "like this" to indicate direct quotes from your manuscript), or elaborate on this item by providing additional information not in the ms, or briefly explain why the item is not applicable/relevant for your study

In the post-treatment questionnaires, both peer counselors and participants had the opportunity to provide qualitative acceptability and feasibility feedback.

DISCUSSION

22) Interpretation consistent with results, balancing benefits and harms, and considering other relevant evidence

NPT: In addition, take into account the choice of the comparator, lack of or partial blinding, and unequal expertise of care providers or centers in each group

22-i) Restate study questions and summarize the answers suggested by the data, starting with primary outcomes and process outcomes (use)

Restate study questions and summarize the answers suggested by the data, starting with primary outcomes and process outcomes (use).

	1	2	3	4	5	
subitem not at all important	○	○	○	●	○	essential

Clear selection

Does your paper address subitem 22-i? *

Copy and paste relevant sections from the manuscript (include quotes in quotation marks "like this" to indicate direct quotes from your manuscript), or elaborate on this item by providing additional information not in the ms, or briefly explain why the item is not applicable/relevant for your study

“The aim of this study was to investigate the efficacy of an adjunctive treatment component that uses task-shifting to enhance engagement in a self-directed, web-based mindfulness intervention for stress and depression among Chinese undergraduate and graduate students. Results indicated that participants assigned to the MIND+ condition showed significantly less attrition and more adherence. In addition, MIND+ participants reported significant improvements in daily ratings of stress and depression across the trial, compared to individuals in the MIND condition. These findings suggest that volunteer peer counselors receiving brief training and weekly supervision may significantly improve participants’ indices of treatment engagement and mental health outcomes in an internet-based mindfulness intervention among college and graduate students in China.

22-ii) Highlight unanswered new questions, suggest future research

Highlight unanswered new questions, suggest future research.

	1	2	3	4	5	
subitem not at all important	○	○	○	●	○	essential

Clear selection

Does your paper address subitem 22-ii?

Copy and paste relevant sections from the manuscript (include quotes in quotation marks "like this" to indicate direct quotes from your manuscript), or elaborate on this item by providing additional information not in the ms, or briefly explain why the item is not applicable/relevant for your study

Future research could explore the mechanisms by which peer support enhances adherence, including factors such as social desirability, expectation, and compliance. Further analyses should explore whether changes in mindfulness mediate the effects of the intervention on depression, anxiety, and stress. Collecting separate data for formal and informal mindfulness practice, or collecting data in real time could improve findings. Future studies can help explore and detect possible dosage effects. Moreover, future research would benefit from having more objective indicators of study, practice, and mindfulness meditation. For example, one patient might report to practice once in a day, but it was a 45-minute body scan, and another patient might report practicing mindfulness 35 times, because they noticed their thoughts or body sensations that many times across the day."

20) Trial limitations, addressing sources of potential bias, imprecision, and, if relevant, multiplicity of analyses

20-i) Typical limitations in ehealth trials

Typical limitations in ehealth trials: Participants in ehealth trials are rarely blinded. Ehealth trials often look at a multiplicity of outcomes, increasing risk for a Type I error. Discuss biases due to non-use of the intervention/usability issues, biases through informed consent procedures, unexpected events.

	1	2	3	4	5	
subitem not at all important	○	○	●	○	○	essential

Clear selection

Does your paper address subitem 20-i? *

Copy and paste relevant sections from the manuscript (include quotes in quotation marks "like this" to indicate direct quotes from your manuscript), or elaborate on this item by providing additional information not in the ms, or briefly explain why the item is not applicable/relevant for your study

Like many e-health trials, neither the researchers nor the participants were blinded in this study.

21) Generalisability (external validity, applicability) of the trial findings

NPT: External validity of the trial findings according to the intervention, comparators, patients, and care providers or centers involved in the trial

21-i) Generalizability to other populations

Generalizability to other populations: In particular, discuss generalizability to a general Internet population, outside of a RCT setting, and general patient population, including applicability of the study results for other organizations

	1	2	3	4	5	
subitem not at all important	○	○	○	◉	○	essential

Clear selection

Does your paper address subitem 21-i?

Copy and paste relevant sections from the manuscript (include quotes in quotation marks "like this" to indicate direct quotes from your manuscript), or elaborate on this item by providing additional information not in the ms, or briefly explain why the item is not applicable/relevant for your study

“The aim of this study was to investigate the efficacy of an adjunctive treatment component that uses task-shifting to enhance engagement in a self-directed, web-based mindfulness intervention for stress and depression among Chinese undergraduate and graduate students. There are several limitations related to this study. First, the sample was small and consisted of a non-clinical sample of English-speaking university students. Before these findings can be generalized, this research should be replicated among participants using the internet-based intervention in their native language and among larger, more diverse samples.”

21-ii) Discuss if there were elements in the RCT that would be different in a routine application setting

Discuss if there were elements in the RCT that would be different in a routine application setting (e.g., prompts/reminders, more human involvement, training sessions or other co-interventions) and what impact the omission of these elements could have on use, adoption, or outcomes if the intervention is applied outside of a RCT setting.

	1	2	3	4	5	
subitem not at all important	○	○	○	◉	○	essential

Clear selection

Does your paper address subitem 21-ii?

Copy and paste relevant sections from the manuscript (include quotes in quotation marks "like this" to indicate direct quotes from your manuscript), or elaborate on this item by providing additional information not in the ms, or briefly explain why the item is not applicable/relevant for your study

In this RCT, all intervention materials were translated from English into Mandarin; in the routine application, the materials are delivered in English. Additionally, the routine application excludes all of the assessment questionnaires utilized in this study, and the standard intervention does not feature peer counselors. “Before these findings can be generalized, this research should be replicated among participants using the internet-based intervention in their native language.”

OTHER INFORMATION

23) Registration number and name of trial registry

Does your paper address CONSORT subitem 23? *

Copy and paste relevant sections from the manuscript (include quotes in quotation marks "like this" to indicate direct quotes from your manuscript), or elaborate on this item by providing additional information not in the ms, or briefly explain why the item is not applicable/relevant for your study

At the commencement of this study, the researchers were under the impression that only large, government-funded trials could be registered in clinicaltrials.gov. It was understood that this study would serve as a pilot to assess the acceptability and feasibility of an internet-based approach for reducing attrition; the researchers did not think to officially register a pilot study. Nonetheless, we are more than willing to register this study post-hoc.

24) Where the full trial protocol can be accessed, if available

Does your paper address CONSORT subitem 24? *

Cite a Multimedia Appendix, other reference, or copy and paste relevant sections from the manuscript (include quotes in quotation marks "like this" to indicate direct quotes from your manuscript), or elaborate on this item by providing additional information not in the ms, or briefly explain why the item is not applicable/relevant for your study

This information is unavailable.

25) Sources of funding and other support (such as supply of drugs), role of funders

Does your paper address CONSORT subitem 25? *

Copy and paste relevant sections from the manuscript (include quotes in quotation marks "like this" to indicate direct quotes from your manuscript), or elaborate on this item by providing additional information not in the ms, or briefly explain why the item is not applicable/relevant for your study

The only source of funding for this study was from the Student Research Award granted by the Center for International Studies at Duke University.

X27) Conflicts of Interest (not a CONSORT item)

X27-i) State the relation of the study team towards the system being evaluated

In addition to the usual declaration of interests (financial or otherwise), also state the relation of the study team towards the system being evaluated, i.e., state if the authors/evaluators are distinct from or identical with the developers/sponsors of the intervention.

	1	2	3	4	5	
subitem not at all important	○	○	○	○	◉	essential

Clear selection

Does your paper address subitem X27-i?

Copy and paste relevant sections from the manuscript (include quotes in quotation marks "like this" to indicate direct quotes from your manuscript), or elaborate on this item by providing additional information not in the ms, or briefly explain why the item is not applicable/relevant for your study

None of the authors or researchers have any conflicts of interest to disclose. Additionally, nobody on the research team has any connection to the developers or sponsors of the “Be Mindful” app.

About the CONSORT EHEALTH checklist

As a result of using this checklist, did you make changes in your manuscript? *

- ○ yes, major changes
- ◉ yes, minor changes
- ○ no

What were the most important changes you made as a result of using this checklist?

Clarification and the use of more precise language in the title; inclusion and elaboration of the informed consent materials.

How much time did you spend on going through the checklist INCLUDING making changes in your manuscript *

10 hours (a lot spent trying to shorten CONSORT responses)

As a result of using this checklist, do you think your manuscript has improved? *

- [x] yes
- [ ] no
- [ ] Other:

Would you like to become involved in the CONSORT EHEALTH group?

This would involve for example becoming involved in participating in a workshop and writing an "Explanation and Elaboration" document

- [ ] yes
- [x] no
- [ ] Other:

Clear selection

Any other comments or questions on CONSORT EHEALTH

Please denote word limits so that I know how long to make responses.

STOP - Save this form as PDF before you click submit

To generate a record that you filled in this form, we recommend to generate a PDF of this page (on a Mac, simply select "print" and then select "print as PDF") before you submit it.

When you submit your (revised) paper to JMIR, please upload the PDF as supplementary file.

Don't worry if some text in the textboxes is cut off, as we still have the complete information in our database. Thank you!

Final step: Click submit !

Click submit so we have your answers in our database!

Submit

Never submit passwords through Google Forms.

This content is neither created nor endorsed by Google. Report Abuse - Terms of Service - Privacy Policy

Google Forms
